# Deciphering principles of nucleosome interactions and impact of cancer-associated mutations from comprehensive interaction network analysis

**DOI:** 10.1093/bib/bbad532

**Published:** 2024-02-07

**Authors:** Wang Xu, Houfang Zhang, Wenhan Guo, Lijun Jiang, Yunjie Zhao, Yunhui Peng

**Affiliations:** Institute of Biophysics and Department of Physics, Central China Normal University, Wuhan 430079, China; Institute of Biophysics and Department of Physics, Central China Normal University, Wuhan 430079, China; Computational Science Program, University of Texas at El Paso, El Paso, TX 79902, USA; Hubei Key Laboratory of Genetic Regulation & Integrative Biology, School of Life Sciences, Central China Normal University, Wuhan 430079, China; Institute of Biophysics and Department of Physics, Central China Normal University, Wuhan 430079, China; Institute of Biophysics and Department of Physics, Central China Normal University, Wuhan 430079, China

**Keywords:** histone/nucleosome interaction, interaction network, histone cancer mutation, nucleosome binding mode, epigenetic regulation

## Abstract

Nucleosomes represent hubs in chromatin organization and gene regulation and interact with a plethora of chromatin factors through different modes. In addition, alterations in histone proteins such as cancer mutations and post-translational modifications have profound effects on histone/nucleosome interactions. To elucidate the principles of histone interactions and the effects of those alterations, we developed histone interactomes for comprehensive mapping of histone–histone interactions (HHIs), histone–DNA interactions (HDIs), histone–partner interactions (HPIs) and DNA–partner interactions (DPIs) of 37 organisms, which contains a total of 3808 HPIs from 2544 binding proteins and 339 HHIs, 100 HDIs and 142 DPIs across 110 histone variants. With the developed networks, we explored histone interactions at different levels of granularities (protein-, domain- and residue-level) and performed systematic analysis on histone interactions at a large scale. Our analyses have characterized the preferred binding hotspots on both nucleosomal/linker DNA and histone octamer and unraveled diverse binding modes between nucleosome and different classes of binding partners. Last, to understand the impact of histone cancer-associated mutations on histone/nucleosome interactions, we complied one comprehensive cancer mutation dataset including 7940 cancer-associated histone mutations and further mapped those mutations onto 419,125 histone interactions at the residue level. Our quantitative analyses point to histone cancer-associated mutations' strongly disruptive effects on HHIs, HDIs and HPIs. We have further predicted 57 recurrent histone cancer mutations that have large effects on histone/nucleosome interactions and may have driver status in oncogenesis.

## INTRODUCTION

Nucleosomes are fundamental structural units of eukaryotic chromatin, which plays a crucial role in packaging and regulating the accessibility of DNA in eukaryotic cell [1]. Nucleosome structure consists of a histone octamer which includes two copies of histone proteins H2A, H2B, H3, and H4, wrapped by about 147 base pairs of DNA molecules [2]. Nucleosomes are central players in chromatin organization and gene regulation [3, 4]. Their interactions with various regulatory proteins such as chromatin remodelers, histone modifiers and transcription factors lead to profound impacts on gene expression and ultimately determine cell fate and identity [4–7]. Most chromatin factors recognize nucleosome structure via multivalent interactions, involving nucleosomal and linker DNA, histone tails, histone globular domains and post-translational modifications (PTMs) [6, 8]. In addition, binding of chromatin factors to nucleosome modulates nucleosome dynamics and conformations, regulating the organization of higher order chromatin strunctures [4, 9]. The binding modes between nucleosome and various chromatin factors and the molecular mechanisms behind these interactions still remain understudied.

Mutations in histone proteins have recently been linked to different types of cancers and many of them have been suggested as ‘oncohistones’ in driving oncogenic procession [10–14]. It has also been suggested that cancer mutations frequently occur on both histone tails and globular domains and have profound effects on nucleosome structure, dynamics and interactions [14–16]. Many oncohistone mutations are located at or near key regulatory PTMs of histone tails, disrupting the reading, writing and/or erasing of these marks [17, 18]. Histone cancer mutations on globular domains, especially for those around acidic patches, can lead to disruptive effects on histone/nucleosome interactions with chromatin factors [15, 16]. Cancer-associated mutations can further disrupt nucleosome stability and dynamics through alteration of histone–histone and histone–DNA binding interfaces, leading to enhanced fundamental chromatin remodeling processes [19]. Currently, thousands of histone cancer-associated mutations have been identified from the clinical sequencing cohorts but their status as driver mutations and molecular effects on nucleosome structure and interaction still remain little known.

In recent years, the number of deposited histone and nucleosome structures have been exponentially increasing with the advances in experimental technique [4]. These structures provide comprehensive information of histone interactions at the atomic level and enable characterizations of histone/nucleosome binding interfaces at atomic resolution [20]. One recent computational study has developed a framework for systematical analysis and classification of nucleosome structures and their complexes [21]. NucPosDB reports various experimental nucleosome maps across different cell types and software for computational analysis of nucleosome positioning and occupancy [22]. In addition, cross-linking mass spectrometry (XL-MS), a powerful experimental approach for identifying protein–protein interactions (PPIs), has been applied to study the histone/nucleosome interactions at a large scale [23–25]. In our recent work, we have constructed human histone interactomes to characterize histone–partner interactions (HPIs) by integrating different types of experimental data [20]. Despite these efforts, a comprehensive mapping of histone/nucleosome interactions still remains a daunting challenge, requiring the integration of different datasets and both computational and experimental approaches.

Histone interaction networks can be one powerful tool to systematically characterize the principles of nucleosome interactions with various chromatin factors and further elucidate the effects of cancer-associated mutations on nucleosome structure and interactions. In this study, we have developed histone interactomes for comprehensive mapping of histone–histone interactions (HHIs), histone–DNA interactions (HDIs), histone-partner interactions (HPIs) and DNA–partner interactions (DPIs). The new histone interactome contains a total of 3808 HPIs from 2544 binding proteins (increased by 6-folds compared with our recent work [20]) and total 581 interactions from histone–histone, histone–DNA and DNA–partner binding across 110 histone variants. With the developed network, we have performed comprehensive analyses on histone binding interfaces, PTM-associated histone interactions, nucleosome–partner binding modes, functionality of histone binding partners and topological properties of networks. Our results have identified preferred binding hotspots on both histone octamer and DNA molecules and unraveled different binding modes between nucleosome and chromatin factors. Last, using the networks, we have performed comprehensive mapping of histone cancer-associated mutations onto histone binding interfaces and quantitatively predicted their effects on different types of histone interactions. Our analyses show that histone cancer mutations lead to strong disruptive effects on HHIs, HDIs and HPIs. We have further identified 57 recurrent histone cancer mutations that strongly affect histone/nucleosome interactions and may have driver status in development of cancers. All data of histone interaction networks and compiled datasets of histone cancer-associated mutations are deposited on GitHub (https://github.com/CCNU-COMPBIO/Histone-Interactome).

## METHODS

### Construction of histone/nucleosome interaction network of different organisms

**Figure 1:**
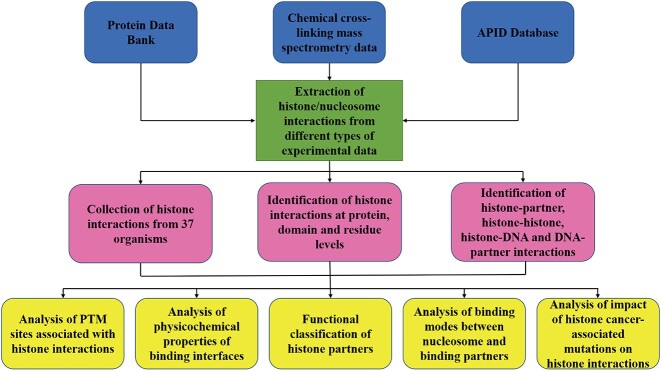
A workflow to construct histone interaction networks at different levels of granularity using PDB structures of histone/nucleosome, cross-linking mass spectrometry experimental data and high-throughput data from the APID database.

We have updated and expanded our previously human histone interaction network [20] with all available nucleosome and histone/nucleosome individual and complex structures in PDB bank [26], recent cross-linking mass spectrometry (XL-MS) experimental data [23, 25] and high-throughput data from APID database [27] (Figure 1). To build the network, we first performed the text search against PDB using a list of keywords associated with histones (Supplementary Table 1). Then, all available structures that include histones and/or any protein binding partners were used for extracting information of histone interactions (Supplementary Tables 2–3). In total, we identified 1208 histone interactions and DNA–partner interactions (DPIs) using 256 individual nucleosome structures, 649 histone complex structures and 234 nucleosome complex structures.

To identify the binding interfaces, we retrieved coordinates of all chains from PDB structures and analyzed their inter-protein and protein-DNA contacts. Histone binding interfaces were identified as the residues located within 5 Å distance between any heavy atoms of histones and their binding partners. Four types of histone interactions were extracted from complex structures including histone–partner interactions (HPIs,partners are the non-histone proteins present in histone/nucleosome complex structures), histone–histone interactions (HHIs), histone–DNA interactions (HDIs) and DNA–partner interactions (DPIs, the interactions between nucleosomal or linker DNA and non-histone proteins). Besides standard protein residues, we have further analyzed the interactions between histone post-translational modified sites and binding proteins. In total, 2886 residue-level histone interactions associated with PTMs were identified from histone/nucleosome structures. Next, similar to our previous work [20], we used the protein domain family annotations from the Conserved Domain Database [28] to identify domain families of histone-binding proteins and construct domain-level network. Details of constructed interactions networks are shown in Supplementary Table 4.

In our structural interaction network, we provided detailed information of both histone and binding partner's interfacial residues including corresponding uniport IDs, interfacial residue number in both PDB structure and protein sequence, residue name, corresponding PDB and chain ID, type of organisms and PTM types. In addition, we also provided information of histone cancer mutations located on any types of the histone interfaces and corresponding predicted impact on histone/nucleosome binding. Residue locations were mapped to the sequences of the corresponding UniprotKB entries using SIFTS [29]. Details of complied histone cancer-associated mutation dataset and predictions of their effects are shown in methods and results sections.

Besides the structural data, we have further constructed histone interaction networks using cross-linking mass spectrometry data from two recent studies [23, 25] (referred to as cross-linking interactome). The cross-linking interactome contains total 1046 interactions from 749 binding proteins (Supplementary Table 4). We have further combined the structural interaction network with cross-linking network, resulting in one integrated histone interactions network including 1355 interactions from 938 binding partners. Furthermore, we have constructed high-throughput interactome using the PPIs identified from high-throughput studies in the APID database, which contains 2963 histone interactions from *Homo sapiens* (Supplementary Table 4). Last, we have constructed the *global histone interactomes* by identifying one additional layer of partners that interact with histone-binding partners (Supplementary Table 5 and Supplementary Figure 1). All source data of histone interaction networks and codes are deposited on GitHub (https://github.com/CCNU-COMPBIO/Histone-Interactome).

### Interactome visualization and topological analysis

All constructed histone interactomes were visualized using Cytoscape [30]. Nodes were annotated with the UniProtKB accession identifiers and the BioPAX_SIF style was used in the network visualization. A degree sorted circle layout was used for visualization of human structural and cross-linking networks at a residue level. The analysis of network topological characteristics was conducted as outlined below. The hub nodes in each histone interaction network were identified using the cytoHubba program [31] by calculating the Maximal Clique Centrality (MCC) score. It is defined as MCC(v) = Σ_C∈S(v)_ (|C|-1)!, where S(v) represents the set of maximal cliques containing node v, and C indicates the size of the maximum clique. A clique is defined as a subset of nodes in an undirected graph where every two distinct nodes are adjacent. Maximal clique is a clique that cannot be extended by including adjacent nodes. Pair-wise comparisons between different networks were performed using the DyNet program [32] and the shared nodes in different networks were identified. Other network topological attributes, including clustering coefficient, topological coefficient, betweenness centrality and node degree, were calculated using the Network Analyzer module in Cytoscape [30].

### Analysis of nucleosome–partner binding modes using nucleosome complex structures

To analyze the binding modes between nucleosome and various binding proteins, we collected all available structures of nucleosome core particles in complex with regulatory proteins from PDB bank [26]. Up to the date of this paper submitted, 234 nucleosome complex structures from 14 types of organisms were available for this study (Supplementary Table 2). For collected nucleosome complex structures, we further removed the redundant nucleosome complex structures (same binding protein with identical nucleosome binding modes) and selected the representative structure for each binding protein using the following scenarios: (i) for each nucleosome binding protein, redundant structures were removed and the structure with the highest resolution and without mutations was selected. (ii) For proteins with multiple binding modes to nucleosome, one structure with highest resolution was selected for each binding mode. (iii) Subnucleosomal structures (lack more than one copy of core histones) were removed. (iv) We excluded the nucleosome structures that have more than 20 base pairs of nucleosomal DNA molecules lost the atomic contacts with histone octamer (5 Å distance was used as threshold). (v) We removed structures with sequence lengths of binding partners less than 10 residues. Last, 83 nucleosome complex structures of 200 nucleosome binding proteins were selected as representative structures and subjected to analysis of nucleosome–partner binding modes (Supplementary Table 6).

To analyze the binding modes between nucleosome and chromatin factors, we characterized the binding interfacial residues on histones and DNA molecules. The histone or DNA interfacial residues were identified as histone residues or DNA molecules with any heavy atoms of non-histone proteins located within 5 Å. Nucleosome complex structures were further categorized into three groups based on their types of binding interfaces: (i) regulatory proteins only interact with nucleosomal or linker DNA; (ii) regulatory proteins only interact with histones; and (iii) regulatory proteins interact with both histone and DNA. To identify the protein binding hotspots on histone octamer and nucleosomal or linker DNA, for each histone residue and DNA base pair, we counted the number of unique nucleosome binding proteins and the mean atomic contact numbers between heavy atoms. We have also performed multiple sequence alignment of human histone variants for each histone type using Clustal Omega 1.2.4 [33] with the default parameters (Supplementary Figures 2–6) and mapped them onto the consensus sequences. Chimera was used for visualization of nucleosome structures [34].

### Functional classifications of histone/nucleosome binding proteins

We performed functional classifications of histone/nucleosome binding proteins using the PANTHER Classification System [35], a comprehensive annotated library of gene family phylogenetic trees. The UniProt IDs of all binding proteins were subjected to the webserver (https://www.pantherdb.org) for functional classification using the PATHER protein class [35, 36]. For identified 83 representative nucleosome complex structures, we have classified them into 20 categories using the functional annotations from NucleosomeDB database [21] (Supplementary Table 7) .

### Collection and analysis of histone cancer-associated mutations

We obtained the histone cancer-associated mutations from cBioportal [37] by querying the curated set of non-redundant studies using a comprehensive set of histone genes compiled from HistoneDB database [38] (Supplementary Table 8). It leads to 8404 histone mutations and corresponding metadata on sequencing and clinical information. We further filtered out targeted sequencing data and samples with the somatic unmatched status [39]. After filtering, 6583 missense mutations from 84 histone genes were obtained across 76 cancer types. We have further compared our collected data with one histone mutation dataset compiled from recent study [15], which has 3860 histone cancer mutations of four core histones after filtering out targeted sequencing data. We observe 37% mutations are shared with the previous dataset (published in 2019) and more than 4000 novel histone mutations have been collected in this study (Supplementary Figure 7). Therefore, we have compiled one combined dataset which integrates those two datasets and contains a total of 7940 histone cancer-associated mutations across 84 histone genes and 83 cancer types (referred to as *combined histone cancer mutation set*).

To mitigate the confounding effects from highly mutated tumors, we filtered out the mutations from samples with a tumor mutation burden of >10 mutations per megabase (Mb) following previous study [15]. We further removed the mutations previously observed in dbSNP database and selected recurrent histone mutations, which are likely to be associated with cancer progression. Recurrent histone cancer mutations are selected as the mutations occurred in at least three different samples in dataset. After filtering, we constructed one refined dataset (referred to as ‘*refined histone cancer mutation set*’) including 891 recurrent histone missense mutations across 60 histone gene and 47 cancer types. Both *combined* and *refined histone cancer mutation set* was subjected to our analysis of effects of mutations on histone/nucleosome structure and interactions.

To elucidate the impact of mutations on histone structure and interactions, we have mapped both *combined* and *refined histone cancer mutation set* onto the human structural histone interaction networks and identified the mutations located on any histone–histone, histone–DNA and histone–partner binding interfaces. To quantify the effects of mutations on histone binding stability, we have calculated the protein binding free energy changes (∆∆Gs) caused by each histone mutation using SAAMBE-3D and SAMPDI-3D approaches [40, 41], which are our recently developed machine learning algorithms to predict the effects of single amino acid mutation on PPIs and protein-DNA interactions. Nucleosome complex structures were subjected to SAAMBE-3D and SAMPDI-3D programs to calculate the ∆∆Gs for each histone interfacial mutation. In the calculation of ∆∆Gs of HHIs and HDIs, histone partners were removed from nucleosome complex structures.

## RESULTS

### Histone interactomes explore histone interactions at different levels of granularities

**Figure 2:**
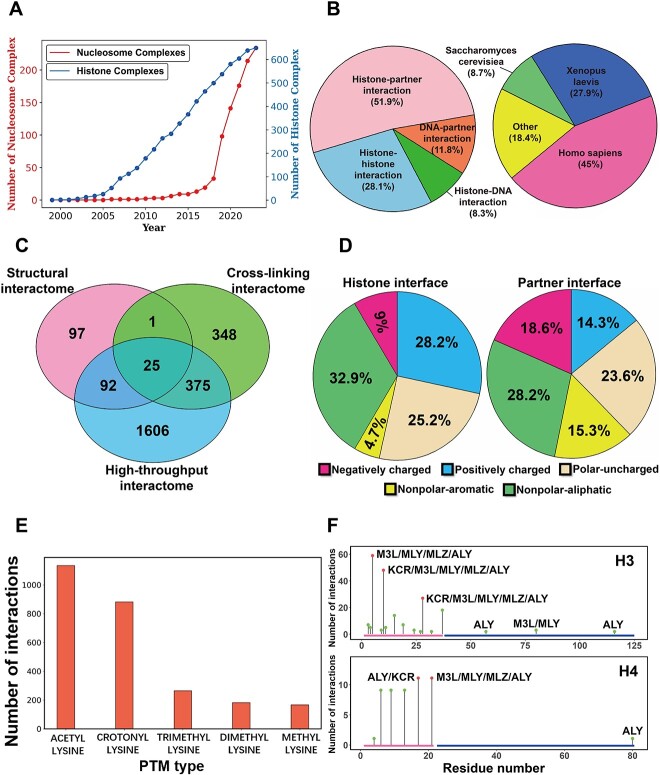
Characterization of histone/nucleosome interactions at a large scale and atomic resolution. (**A**) Number of deposited histone and nucleosome complex structures in PDB bank per year. (**B**) Number of identified histone interactions per interaction type (left) and per organism (right). (**C**) Comparison of the histone binding proteins among human structural, cross-linking and high-throughput interactomes. The histone binding partners observed in at least two different interactomes and their functional classifications are shown in Supplementary Table 14 and Supplementary Figure 14. (**D**) Analysis of physicochemical properties of the binding interfaces of histone (left) and histone binding partners (right). Interfacial residues are categorized into five groups based on their physicochemical properties including polar-uncharged, nonpolar-aliphatic, nonpolar-aromatic, negatively charged and positively charged residues. (**E**) Number of associated HPIs (residue-level) per histone PTM type. (**F**) Number of associated HPIs per histone modification site mapped onto the consensus sequences of histone H3 and H4. The consensus sequences were taken from multiple sequence alignment of histone variants. The histone tail and globular regions are indicated by red and blue bars at the bottom. The PTM sites in histone protein are shown with green circles, where sites involving number of histone interactions more than 20 in histone H3 and more than 10 in histone H4 are highlighted in red. The analysis of other histone types is shown in Supplementary Figure 13.

**Figure 3:**
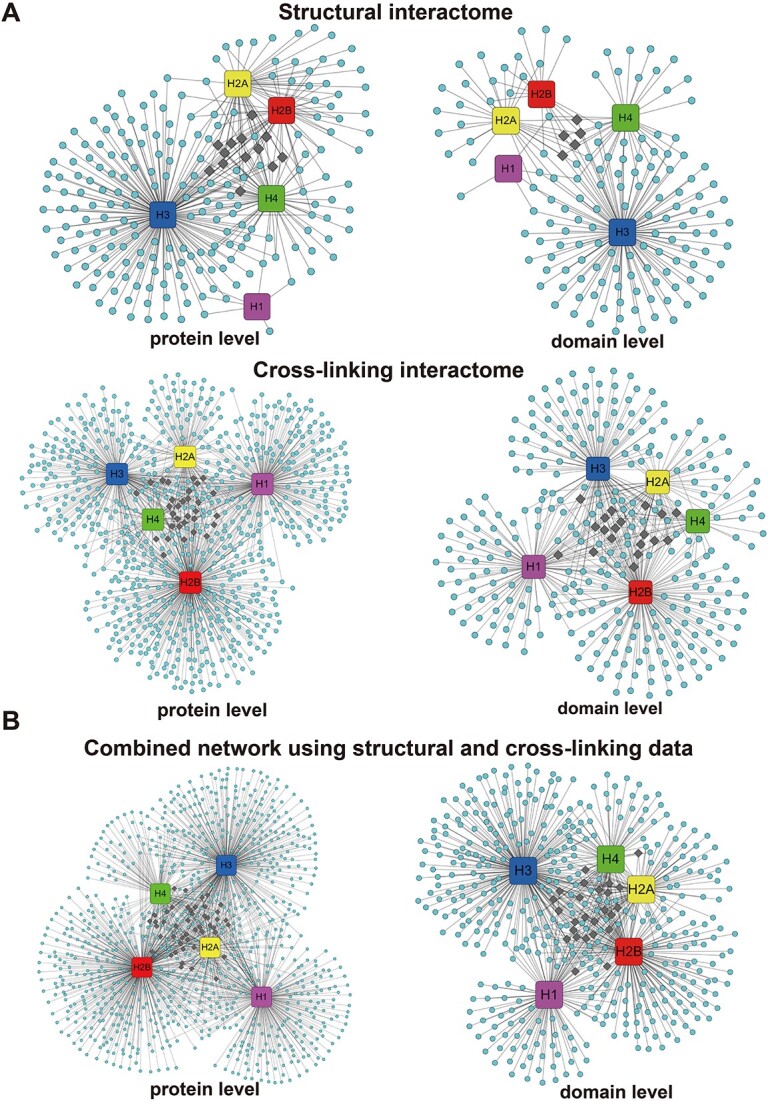
Histone interactomes explore histone interactions at different levels of granularities. (**A**) Human structural and cross-linking interactomes are constructed at different granularity levels. The protein- and domain-level networks are shown as preferred layout in Cytoscape. Histone H1, H2A, H2B, H3 and H4 are colored as purple, yellow, red, blue and green while binding partners are shown in cyan. The hub nodes in networks (MCC score ≥ 4) are highlighted as dark grey. (**B**) Combined human histone interaction network using structural and cross-linking data. The hub nodes in networks (MCC score ≥ 4) are highlighted in dark grey. Biases in histone interactions are mitigated in the combined network. Identified hub nodes are shown in Supplementary Tables 9 and 10.

Through the advances in experimental techniques, the numbers of deposited histone and nucleosome structures have been exponentially increasing in recent years (Figure 2A). Up to the date of this paper submitted, there have been 649 histone complex structures and 234 nucleosome complex structures available in PDB bank, almost increased by 7.5-folds compared with the numbers in 2018 (Figure 2A, Supplementary Figure 8 and Supplementary Table 2). These structures can provide comprehensive information of histone interactions at the atomic level and physical-chemical properties of binding interfaces, which is essential for us to understand the principles of nucleosome interactions. Herein, we have constructed new histone interaction networks for comprehensive mapping of HHIs, HDIs, HPIs and DPIs using different experimental data. The developed histone interactomes contain a total of 3809 HPIs from 2544 binding proteins and 339 histone–histone, 100 histone–DNA and 142 DNA–partner binding across 110 histones. We have further explored different types of histone interactions at three levels of granularities: protein-, domain- and residue-level (Figure 3A, Supplementary Figure 9 and Supplementary Table 4).

Using all available structures, we have identified histone interactions from 37 types of organisms. Majority of histone interactions were extracted from human histone/nucleosome structures, followed by *Xenopus laevis* and *Saccharomyces cerevisiae* (Figure 2B, Supplementary Figure 10 and Supplementary Table 11). Four types of histone/nucleosome interactions were considered in our networks including HPIs, HHIs, HDIs and DPIs, where partners represent the non-histone proteins that interact with histone/nucleosome. In constructed histone interaction networks, HPIs are the most profound interaction types and account for 51.9% histone interactions, then followed by HHIs and DPIs (Figure 2B and Supplementary Table 12). The HHIs and HDIs are observed across various histone variant types and indeed we have identified HHIs and HDIs from 110 types of histone variants (Supplementary Table 13).

Besides the structural interactome, we have also constructed the human cross-linking and high-throughput interactomes using the XL-MS data and APID database (Figure 3A and Supplementary Figure 11). The cross-linking interactome contains 1046 human histone interactions (protein-level) from 749 binding proteins, while 2963 histone interactions from 2098 binding proteins were included in high-throughput interactome. To analyze the similarities and differences of three networks, we have further quantitatively compared the histone binding proteins between structural, cross-linking and high-throughput interactomes. There are only a relatively small portion of binding proteins shared among three networks, especially for structural and cross-linking interactomes (Figure 2C, Supplementary Figure 12 and Supplementary Table 14). Indeed, the structural interaction network has majority of interactions from histone H3 and H4, while most interactions in cross-linking interactome are from H1 and H2B (Figure 3A). The reason could be that histone H1 and H2B have higher lysine contents than other histones and chemical cross-linking mass spectrometry experiments are based on Lys-Lys chemical crosslinks. In addition, PDB database contains lots of complex structures of H3 and H4 tails with PTM readers, writers and erasers but very few H1 interactions are present since long disordered N- and C-terminal regions of histone H1 lead to difficulties in experimental structural characterization. Since these two networks highly complement each other, we can combine structural and cross-linking interactomes and this leads to one combined network including 1355 interactions from 938 proteins. The combined network gives a comprehensive representation of HPIs at the atomic level and the biases in histone interactions are greatly mitigated (Figure 3B and Supplementary Table 4).

### Histone interactomes enable systematic analysis of histone interactions at a large scale

With the developed histone interactomes, we have performed systematical analyses on histone PTM site-partner binding, physicochemical properties of histone/nucleosome binding interfaces, functionality of binding proteins, nucleosome–partner binding modes (discussed in the next section) and topological properties of interaction networks (Figure 1). First, we have analyzed the physicochemical properties of the binding interfaces of histone proteins and binding partners (Figure 2D and Supplementary Table 15). We observe that the positively charged amino acids predominate at the binding interface of histones and the negatively charged interfaces are relatively minor although acidic patches are well-defined nucleosome binding hotpots (Figure 2D). For the binding interfaces of the histone binding partners, the positively charged residues are less abundant compared to the negative ones (Figure 2D). It suggests the complements of residue net charges in binding interfaces and existences of strongly favorable electrostatic interactions between histone and binding partners. It should also be noted that the charge complementarity of histone binding interfaces exists not just around the well-characterized negative acidic patches but also on other histone globular regions with positive charges.

**Figure 4:**
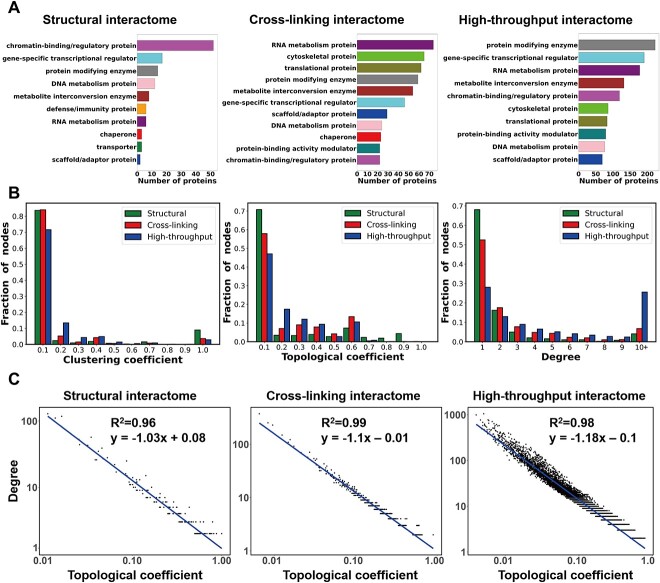
Functional and topological analysis of histone interaction networks. (**A**) Functional classification of histone-binding proteins in human histone interactomes. Proteins were classified using PANTHER protein classes (top 10 ranked protein classes are shown). (**B**) Analysis of topological properties of structural, cross-linking, and high-throughput global interactomes. In all three networks, a strong power-law decay of topological coefficient and clustering coefficient (Supplementary Figure 15) values was observed with the increases in the node degree.

Second, we have identified the histone PTM sites associated with HPIs from histone/nucleosome complex structures. Histone lysine acetylation, lysine crotonylation and lysine trimethylation are the modifications involving most HPIs in available structures (Figure 2E and Supplementary Table 16). Histone H3 and H4 harbor the majority of histone modifications and the modified sites in tails regions are the binding hotspots targeted by various readers, writers and erasers such as Lysine-specific demethylase 4A, E3 ubiquitin-protein ligase UHRF1, PHD finger protein 1 and Histone-lysine N-methyltransferase 2A (Figure 2F and Supplementary Figure 13). Interestingly, H3K56ALY, H3K79M3L/ALY, H3K115ALY and H4 K79ALY are located on histone globular domains, which also participate in different HPIs although less frequent than modified tail residues (Figure 2F). Next, using the PANTHER classifications, we further analyzed the biological functions of histone binding proteins. Interestingly, we find that despite few overlappings among three networks, the biological functions of binding proteins in structural, cross-linking and high-throughput networks are similar and the top ranked classes are chromatin regulatory proteins, DNA/RNA metabolism proteins, gene-specific transcriptional regulators and protein modifying enzymes (Figure 4A, Supplementary Figure 14 and Supplementary Tables 17–19).

Last, we have performed topological analysis of histone interactomes and identified the binding proteins as hubs in the network. To do so, we have identified one additional layer of partners that interact with histone-binding proteins and built the *global histone interactomes* for structural, cross-linking and high-throughput networks (Supplementary Figure 1 and Supplementary Table 5). We have analyzed the topological properties of *global interaction networks* by calculating the degree, clustering coefficient, topological coefficient and MCC scores. It is shown that the majority of nodes exhibit low clustering and topological coefficients and have coefficient values less than 0.1 (Figure 4B). Similar to our previous results [20], the values of the topological and clustering coefficient of the nodes follow a strong power-law decay with the increases of the degree of node, suggesting the histone interactome as a scale-free network (Figure 4C and Supplementary Figure 15). In histone interaction networks, the majority of nodes are sparsely connected while only a small subset of proteins act as hubs that play essential roles in chromatin regulatory processes. To identify the hub nodes, we have ranked all histone binding proteins by the MCC scores and a total of 41 and 47 hub proteins are identified in the global structural and cross-linking networks (Supplementary Tables 20–21), which involves the most histone/nucleosome-associated interactions and could play essential roles in epigenetic regulation. We have further performed functional classifications of hub proteins for global structural and cross-linking networks (Supplementary Tables 22–23). On the top of the list, majority of hub proteins belong to translational protein (proteins involved in translation of mRNA to protein), chaperone, defense/immunity protein, gene-specific transcriptional regulator, cytoskeletal protein, RNA metabolism protein and protein modifying enzyme.

### Regulatory protein reveals preferred binding hotspots on both nucleosomal/linker DNA and histone octamer

**Figure 5:**
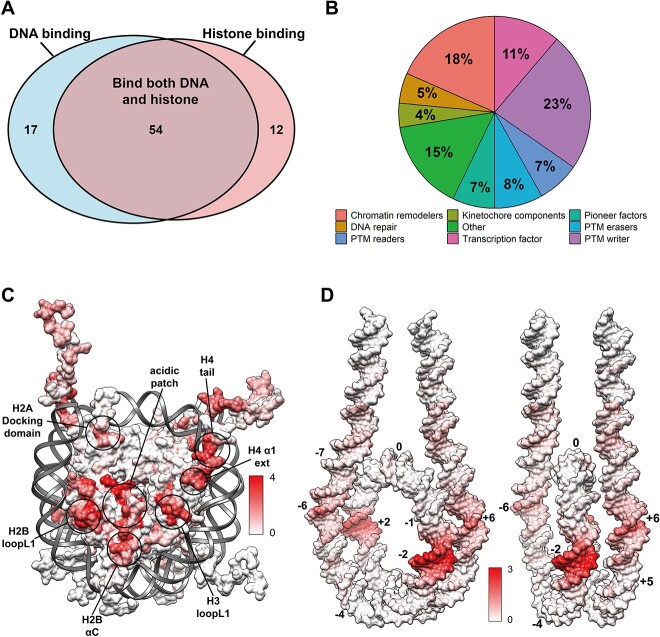
Characterization of binding hotspots in histone proteins and DNA molecules within nucleosome. (**A**) Classification of nucleosome complex structures by their binding modes. (**B**) Classification of nucleosome complex structures by the function of binding partners. The functional annotations used for structural classification are taken from the NucleosomeDB database. (**C**) The number of binding proteins per residue mapped onto histone octamer within the nucleosome structure. The nucleosome representation is generated from PDB 1KX5. (**D**) The number of binding proteins per DNA base pairs mapped onto nucleosomal and linker DNA within the nucleosome structure. The DNA representation is generated from PDB 7K5X and histone octamer is no shown.

To analyze the binding modes between chromatin factors and nucleosome, we first performed classifications of nucleosome complex structures based on their locations of binding interfaces on histones or DNA molecules (Figure 5A). Our results indicate that majority of binding proteins interact with both histone and nucleosomal or linker DNA via a multivalent binding mode, while there are only 20.5% and 14.5% partners bind histones or DNA molecules alone (Supplementary Table 24). Using 83 representative nucleosome complex structures (see Methods about how those structures are selected), we analyzed the binding hotspots of different chromatin factors on histone octamer and DNA molecules. To do so, we mapped the binding sites onto the histones or DNA molecules within nucleosomes and counted the number of unique nucleosome binding proteins per histone residue or DNA base pair (Figure 5). Our analysis has identified multiple binding hotspots on histone octamer including acidic patch, H2A docking domain, H2BloopL1, H2BαC, H3 loopL1 and H4 α1 ext. It should be noted that acidic patch and H3 loopL1 are negatively charged globular regions, while other hotspot regions are generally dominated by positive charges.

Besides the histone octamers, we have also characterized the preferred binding regions on nucleosomal and linker DNA by different chromatin factors (Figure 5 and Supplementary Figure 16). Interestingly, our results show that chromatin factors recognize nucleosomes via several preferred DNA regions mostly around SHL ±2, ±6 and linker DNA. Most chromatin factors bind nucleosome through multivalent binding with both histone and DNA and histone tail H3 and H4 are also likely involved. In contrast, the DNA regions near the dyad and SHL ±3 to ±4 are the regions least targeted by binding proteins. Indeed, our previous simulations have indicated that these DNA regions are extensively bound with histone tails and undergo decreased solvent accessibility [42].

Pioneer factors are critical nucleosome binders and specifically recognize nucleosomal DNA to induce the chromatin opening [7, 43, 44]. Here, we have further analyzed the binding modes of several known pioneer factors with nucleosome including SOX2, SOX11, OCT4, CBF1 and P53. Although pioneer factors have been previously suggested to mainly target entry/exit of nucleosomal DNA, we show that pioneer factors can have different binding modes with nucleosome structures and indeed target multiple DNA regions such as SHL ±2, ±5, ±6 and linker DNA (Supplementary Figure 17). This is also supported by our recent large-scale analysis of nucleosome-pioneer factor binding profiles using ChiP-seq and MNase-seq data [44].

**Figure 6:**
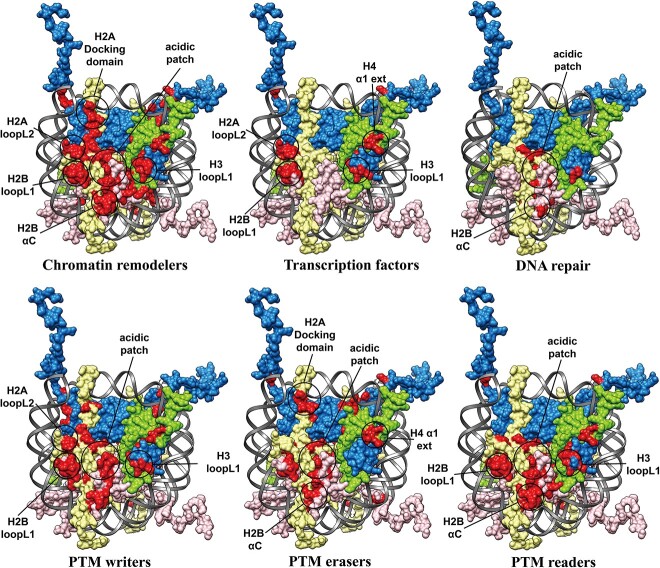
Comparison of binding modes of nucleosome–partner interactions across different functional types. Binding interfaces on histone octamers are marked as red and histone residues that interact with at least two different partners are highlighted with circles. Histones H2A, H2B, H3 and H4 are colored as yellow, pink, blue and green, respectively. The nucleosome representation is generated from PDB 1KX5.

Last, using the annotations from NucleosomeDB database [21], we have categorized all nucleosome complex structures into 20 types based on the functions of nucleosome binding partners and then compared their binding modes across different functional types (Figure 6). Our results indicate that among the different functional classes, chromatin factors target nucleosome through both common and unique binding hotspots. For example, although acidic patches are shared binding sites for most partners, binding of transcription factors only involves the H2 BloopL1, H2A loopL2, H3 loopL1 and H4 α1 ext. Chromatin remodelers interact with the largest regions of histone octamers while DNA repair proteins have relative minor binding sites, mostly around acidic patch. Acidic patch, H2B loopL1, H2B αC and H4 α1 ext are shared binding regions for PTM reader, writer and eraser.

### Interaction network analyses reveal the disruptive effects of cancer mutation on histone/nucleosome interactions

Mutations in histone proteins are associated with various types of cancers and have profound effects on histone/nucleosome structure, dynamics and interactions [10–13]. However, despite existences of thousands of cancer-associated histone mutations in databases, their molecular effects on histone/nucleosome structure and function, especially on histone/nucleosome interactions, still remain understudied. At a large scale and atomic resolution, there is still lack of computational and experimental studies to systematically analyze the effects of histone cancer mutations on nucleosome stability and interactions. Taking advantage of constructed histone interaction networks, we have mapped 7940 unique histone cancer-associated mutations onto total 419,125 HPIs, HHIs and HDIs at residue level and atomic resolution. Then, we quantitatively characterized their impact on histone interactions and binding interfaces.

Through exploring whole exome/genome sequencing data from cBioPortal [37], we have collected 8404 histone cancer mutations across 86 histone types (see methods for more details). We observe 37% of mutations are shared with one dataset published in 2019^15^ and more than 4000 novel histone mutations have been collected in this study (Supplementary Figure 7). Therefore, we complied one combined mutation set which integrates those two datasets and contains total 7940 cancer-associated histone mutations across 84 histone genes and 83 cancer types (referred to as *combined histone cancer mutation set*). In addition, we have further compiled one *refined histone cancer mutation set*, which contains 891 recurrent cancer mutations across 60 histone genes and 47 cancer types (see methods for details).

**Figure 7:**
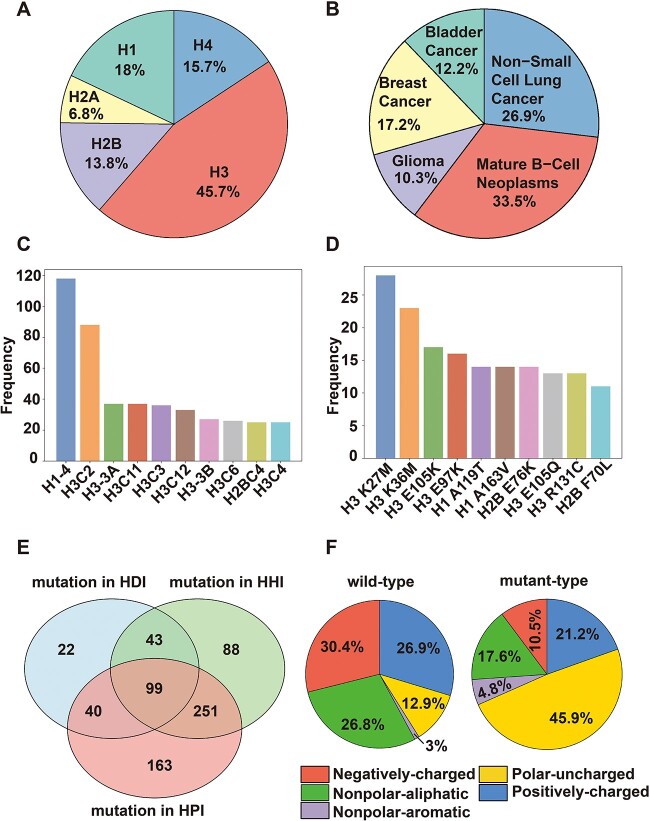
Mapping of histone cancer-associated mutations onto human structural histone interaction network. (**A**) Percentage of recurrent histone cancer mutations per histone type. (**B**) Percentage of recurrent histone cancer mutations per cancer type. (**C**) Rank of histone genes by their carried number of recurrent cancer mutations. (**D**) Rank of histone cancer mutations by their occurrences. (**E**) The number of recurrent histone cancer mutations mapped onto HHI, HDI and HPI networks. (**F**) Analysis of physicochemical properties of histone binding interfaces in both wild-type (left) and mutant-type (right). Mutations from the refined histone cancer mutation set were used for the analyses in this figure.

Histone H3 harbors most recurrent cancer mutations and then followed by histone H1 and H4 while the least recurrent mutations were observed in histone H2A (Figure 7A). The top prevalent cancer types observed in our complied datasets are Mature B-cell Neoplasms, Non-small cell lung cancer, Melanoma, Colorectal cancer and Breast cancer, accounting for more than half of patients (Figure 7B and Supplementary Figure 18B). Next, we have further identified the most frequently mutated histone genes and histone mutations with the highest recurrences in cancer patients (Figure 7 and Supplementary Figure 18). Interestingly, histone gene H1-4 harbors the highest number of histone cancer mutations. Indeed, many recent studies have indicated the pathogenic relevance of histone H1-4 mutations in cancers and their high recurrences in particular cancer types such as lymphomas [45–47]. A very recent study has shown that H1 mutations can result in a profound architectural remodeling of the genome, characterized by large-scale shifts of chromatin from a compacted to a relaxed state [45]. Our analyses have also captured many known histone driver mutations such as H2BE76K, H3K27M, H3K36M and H3G34R, which could help to identify other unknown potential drivers.

To elucidate the effects of cancer-associated mutations on histone/nucleosome interactions, we systematically mapped both *refined* and *combined histone mutation sets* onto the histone interaction networks and identified the mutations which have the disruptive effects on histone binding. In total, 1727, 3578 and 3082 mutations in *combined sets* (Supplementary Figure 18e) and 204, 481 and 553 recurrent mutations in *refined set* (Figure 7E) were mapped onto the histone–DNA, histone–histone and histone–partner binding interfaces, respectively. Interestingly, we have identified 99 recurrent histone mutations in *refined mutation set* (880 mutations in *combined mutation set*) located on all three types of histone binding interfaces, which may lead to highly profound effects on nucleosome structure, interactions and dynamics. Through mapping partner binding hotspots and cancer mutation hotspots onto histone consensus sequences from multiple sequence alignments, we observed a considerable overlapping between mutation hotspots and histone partner binding hotspots (Supplementary Figure 19). Indeed, more than 50% of binding hotspots in core histones carry at least five different cancer-associated mutations, indicating the enrichment of cancer mutations on histone binding interfaces (Supplementary Table 25). Moreover, our analysis on PTM sites also reveals the extensive overlapping between histone PTM sites and cancer mutation hotspots (Supplementary Table 26).

**Figure 8:**
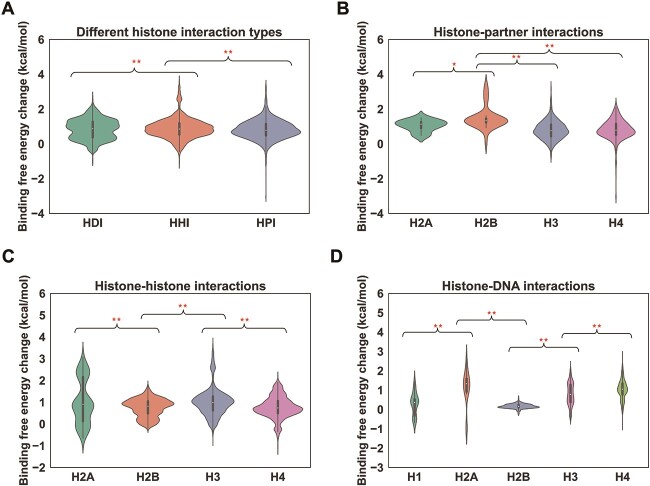
Analysis of the impact of histone mutations on different types of histone interactions. (**A**) Distributions of binding free energy changes (∆∆Gs) caused by recurrent histone cancer mutations located on different types of histone binding interfaces. (**B**), (**C**) and (**D**) Distributions of ∆∆Gs caused by recurrent histone cancer mutations in HPIs, HHIs and HDIs per histone type. Binding free energy change (∆∆G) values were calculated for each histone mutation that can be mapped onto the human histone structural interaction network. Mutations from the refined set were used for the calculation in this figure. Tukey HSD tests were performed to compare the differences of ∆∆G value distributions and the null hypothesis is that the mean values of ∆∆Gs in two groups are equal. ^*^P-value <0.05; ^*^^*^P-value <0.005.

Next, we have asked how the histone cancer mutations can alter the physicochemical properties of histone binding interfaces. To do so, we have first characterized the physicochemical properties of the interfacial residues in both wild type and mutant type (Figure 7F and Supplementary Figure 18F). Our analyses indicate that histone cancer-associated mutations generally suppress both positive and negative charges on histone binding interfaces and at the same time, increase the number of uncharged and hydrophobic residues. Those changes would greatly disrupt different types of histone interactions. To validate our hypothesis, we have further quantitatively calculated the change of binding free energies (∆∆Gs) upon histone mutations for each interaction type (Figure 8 and Supplementary Figure 20). It is shown that histone cancer-associated mutations have strongly disruptive effects on HHIs, HDIs and HPIs, with median decrease of binding free energies of about 1 kcal/mol. In HPIs, mutations in H2A and H2B have the largest destabilizing effects and then followed by histone H3 and H4 (Figure 8 and Supplementary Table 28). In HDIs, mutations in histone H2A and H4 overall lead to the largest changes in binding energies while the effects of H1 and H2B mutations are relatively minor. The distributions of ∆∆Gs are relatively similar among different histone types in HHIs.

**Figure 9:**
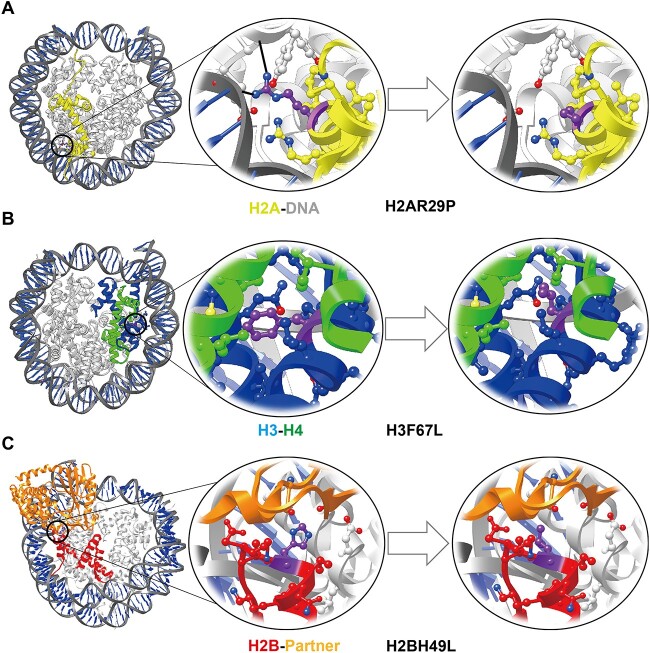
Representative recurrent histone cancer mutations with strongly disruptive effects on histone interactions. (**A**) H2AR29P, (**B**) H3F67L and (**C**) H2BH49L strongly destabilize HHIs, HDIs and HPIs. PDB structures 7U51, 6JR1 and 7JO9 are used to generate the nucleosome representation. H2B-partner interactions show the impact of H2BH49L mutation on the binding of Cyclic GMP-AMP synthase to the nucleosome. Mutation sites are highlighted in purple. Histone H2A, H2B, H3 and H4 are colored as yellow, red, blue and green, respectively, and nucleosome binding partner is shown in orange.

Last, we have identified 57 recurrent histone cancer mutations (occurred in at least three different samples) that can cause substantially destabilizing effects on histone–histone, histone–DNA and histone–partner binding (Figure 9 and Supplementary Table 29-31). In HDIs, we have identified 23 mutations that cause at least 1 kcal/mol decrease in histone–DNA binding free energies (Supplementary Table 29). Majority of these mutations are located on the Arg and Lys residues anchored onto the DNA major or minor grooves within nucleosome structures. It should also be noted that we have identified seven mutations (H3R116P, H3R40C, H3K36M, H3R53H, H3E50D, H3R52C, H3Y41C) that are near the entry/exit of nucleosomal DNA and may affect the DNA dynamics such as unwrapping or breathing. Moreover, we have characterized 34 recurrent cancer mutations that are located on histone–histone binding interfaces and strongly destabilize the histone–histone binding (mean ∆∆G > = 1.5 kcal/mol; Supplementary Table 30). Among these mutations, five mutations affect H2A-H2B interfaces and 29 of them are for H3–H4 interfaces. Those mutations could affect the stability of nucleosome structure by disrupting the histone interfaces on octamer (Figure 9). Last, 23 recurrent cancer mutations were identified to strongly disrupt histone/nucleosome–partner interactions (mean ∆∆G > = 1.5 kcal/mol; Supplementary Table 31). These mutations are located on both histone globular domain and tails. Various histone/ nucleosome–partner interactions such as Kinetochore components, RNA polymerases, DNA repair proteins, PTM readers, writers and erasers were strongly affected. Last, we have found the most prevalent cancer types to these mutations and Breast Cancer, Bladder Cancer and Non-Small Cell Lung Cancer are ranked at the top (Supplementary Figure 21).

## DISCUSSION

Mapping of protein interaction networks related to chromatin with high resolution is a daunting task due to their dynamic transient nature and involvement of both DNA and proteins via multivalent interactions. Through the integration of diverse data including structural, cross-linking and high-throughput data, we constructed new histone interactomes for comprehensive mapping of HHIs, HDIs, HPIs and DPIs for 37 organisms. The new histone interactome contains a total of 3808 HPIs, increased by 6-folds compared with our recent work [20]. In this study, we still observed an underrepresentation of HPIs for certain histone types in structural and cross-linking networks, which arised from the biases in available experimental data. Those two networks indeed have few overlappings and highly complement each other. The constructed combined interactome by integration of structural and XL-MS data can greatly reduce the imbalance in current experimental datasets and give more complete picture of histone/nucleosome interactions at a large scale and atomic resolution (Figure 3B).

Histone interaction networks enable us to perform systematic analysis on histone/nucleosome interactions. In this study, large-scale analyses were performed for different aspects of histone/nucleosome interactions including histone PTMs-partner binding, physicochemical properties of binding interfaces, functionality of histone binding proteins, nucleosome–partner binding modes and topological properties of interaction networks. We observed a strong complement of residue net charges in binding interfaces, which not just exist around the well characterized acidic patches but also on other histone globular regions with positive charges, pointing to the critical roles of electrostatics in modulation of chromatin structures and related interactions [48–51]. We have also identified top ranked classes of proteins in histone binding and the hub nodes in the histone interactome, mostly from chromatin regulatory proteins, DNA/RNA metabolism proteins, gene-specific transcriptional regulators and protein modifying enzymes (Supplementary Tables 22 and 23). Our results underscore the complexity of histone interactions in gene expression, DNA repair, and epigenetic regulation and identifications of specific hub proteins could provide potential targets for chromated-related diseases [52–54]. In this study, we have also unraveled the binding modes between nucleosome and different classes of binding partners. Our results indicate that besides acidic patch, multiple regions on histone octamer can be targeted by chromatin factors via multivalent binding. Preferred DNA regions by chromatin factors have also been revealed on nucleosomal and linker DNA, mostly around SHL ±2, ±6 and linker DNA. Such preferences on the DNA binding sites may be related to histone tail interactions [8, 42]. Our results have further unraveled diverse binding modes between pioneer factors and nucleosome structures and this is supported by recent experimental and computational studies [44, 55–57].

The impact of histone cancer-associated mutations on histone/nucleosome structure still remains understudied. There is still lack of computational or experimental studies to systematically characterize the effects of those mutations on nucleosome structure and interactions at a large scale and atomic resolution. In this study, we have performed a comprehensive mapping of 7940 unique histone cancer-associated mutations onto a total of 419,125 HPIs, HHIs and HDIs at the residue level. Quantitative analyses of their impact on histone interactions and binding interfaces were further performed. Our large-scale analyses point to mutations' strong disruptive effects on HHIs, HDIs and HPIs and suppression of charges on histone-binding interfaces. Those alterations on electrostatics may not only affect the histone/nucleosome interactions but also the high-order chromatin structures. Our analyses are able to capture many known histone driver mutations and we have also predicted 57 novel recurrent histone cancer mutations, which strongly affect histone/nucleosome interactions and may have potential driver status. These mutations should be subjected to deeper exploration of the biological context and functional consequences. It may also inform the development of targeted therapies and personalized medicine approaches for cancer treatment.

**Key Points**

- Histone interactomes were developed for comprehensive mapping of histone–histone, histone–DNA, histone–partner and DNA–partner interactions.
- Histone/nucleosome interactions at different levels of granularities (protein-level, domain-level and residue-level) were characterized at a large scale and atomic resolution.
- Our analyses have identified the preferred binding hotspots on both nucleosomal/linker DNA and histone octamer and revealed diverse binding modes between nucleosome and binding partners.
- We have characterized the effects of histone cancer mutations on histone–histone, histone–DNA and histone–partner interactions, and predicted 57 recurrent cancer mutations that have large disruptive effects on histone/nucleosome interactions and may have driver status in development of cancers.

## Supplementary Material

Supplementary_material_final_bbad532_V5

## Author Biographies

**Wang Xu** is a graduate student at the Institute of Biophysics and Department of Physics in Central China Normal University.

**Houfang Zhang** is a graduate student at the Institute of Biophysics and Department of Physics in Central China Normal University.

**Wenhan Guo** is a graduate student at the Computational Science Program in University of Texas at El Paso, USA.

**Lijun Jiang** is a professor at the School of Life Sciences in Central China Normal University. Her research interests are design and development of functional probes or materials, especially for the achievement of responsive fluorescence and interference on biological targets.

**Yunjie Zhao** is a professor at the Institute of Biophysics and Department of Physics in Central China Normal University. His research interests are computational biophysics and bioinformatics.

**Yunhui Peng** is an associated professor at the Institute of Biophysics and Department of Physics in Central China Normal University. His research interests are computational biophysics and bioinformatics.
