# Supplementary material for: Deciphering principles of nucleosome interactions and impact of cancer-associated mutations from comprehensive interaction network analysis
Source: Brief Bioinform. 2024 Feb 7;25(2):bbad532. doi: 10.1093/bib/bbad532 (PMC10851104; doi:10.1093/bib/bbad532)
Supplement: Supplementary_material_final_bbad532_V5 [file supplementary_material_final_bbad532_v5.docx]

**Supplementary Figure 1.** (a) One representative figure to show proteins that interact with histone-binding partners are identified (orange nodes) and added as one additional layer to the initial networks (green and pink nodes) and *histone global interactomes* are constructed. (b), (c) and (d) are the human structural, cross-linking and high-throughput *global histone interactomes.* Histone H1, H2A, H2B, H3 and H4 are colored as purple, yellow, red, blue and green while binding proteins are shown in pink.


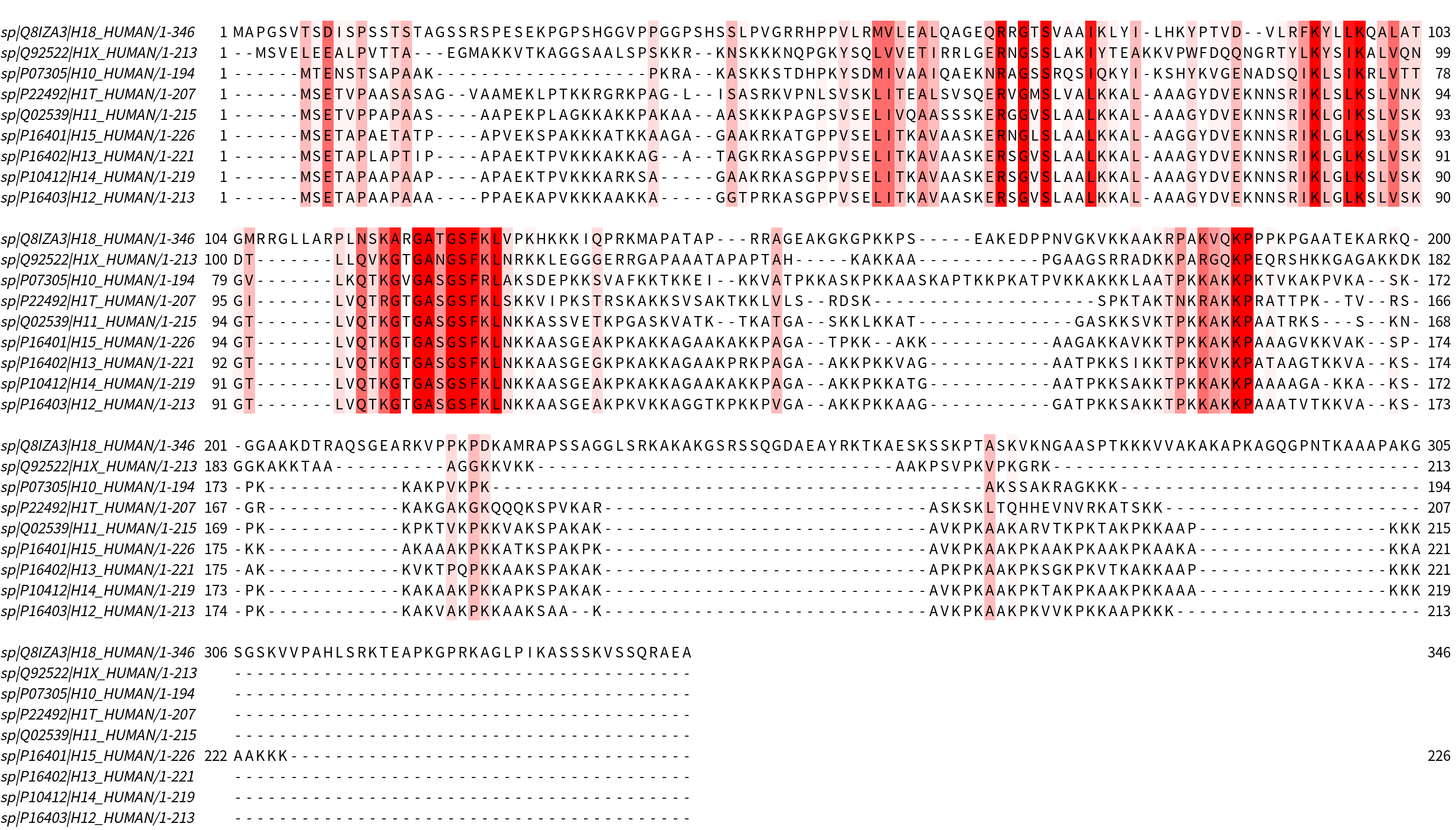


**Supplementary Figure 2.** Multiple sequence alignment of human histone H1 variants in histone interaction network. The sequences are extracted from UniProt^1^ and HistoneDB 2.0^2^ and aligned using Clustal Omega 1.2.4^3^. Conserved sites are shown in red.


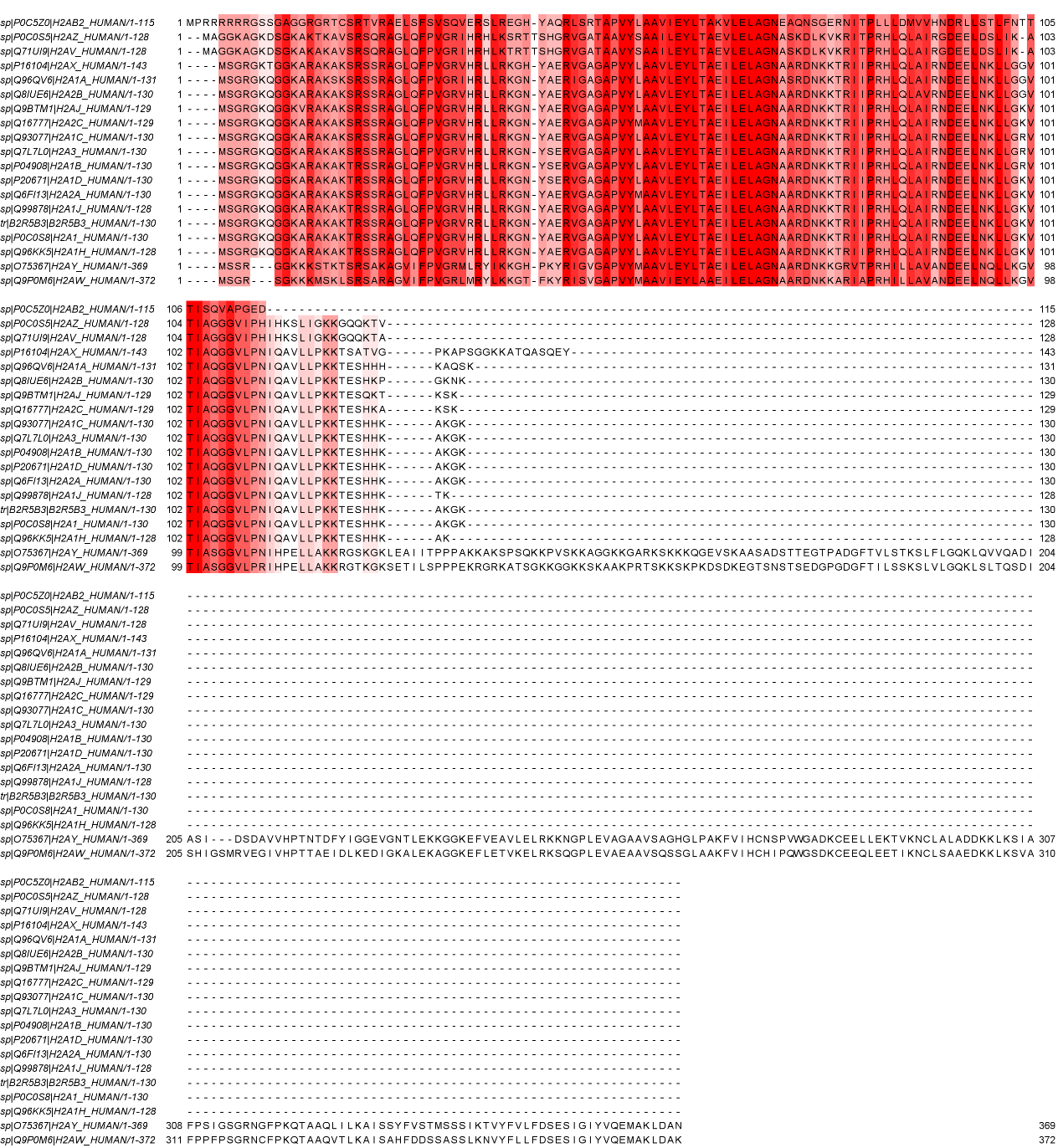


**Supplementary Figure 3.** Multiple sequence alignment of human histone H2A variants in histone interaction network. The sequences are extracted from UniProt^1^ and HistoneDB 2.0^2^ and aligned using Clustal Omega 1.2.4^3^. Conserved sites are shown in red.


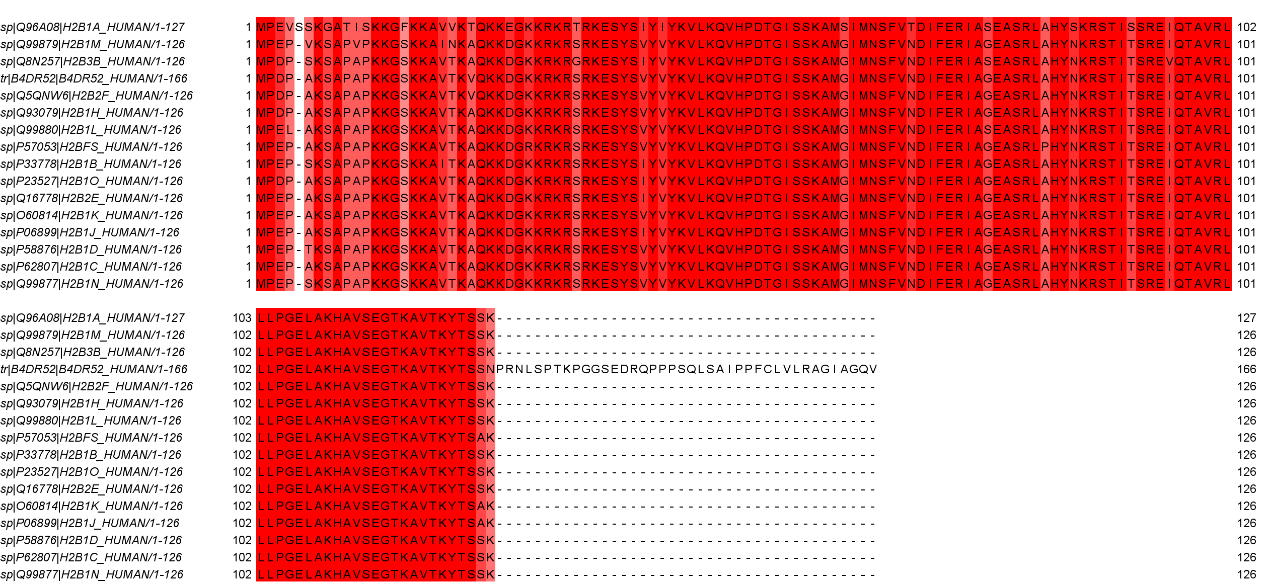


**Supplementary Figure 4.** Multiple sequence alignment of human histone H2B variants in histone interaction network. The sequences are extracted from UniProt^1^ and HistoneDB 2.0^2^ and aligned using Clustal Omega 1.2.4^3^. Conserved sites are shown in red.


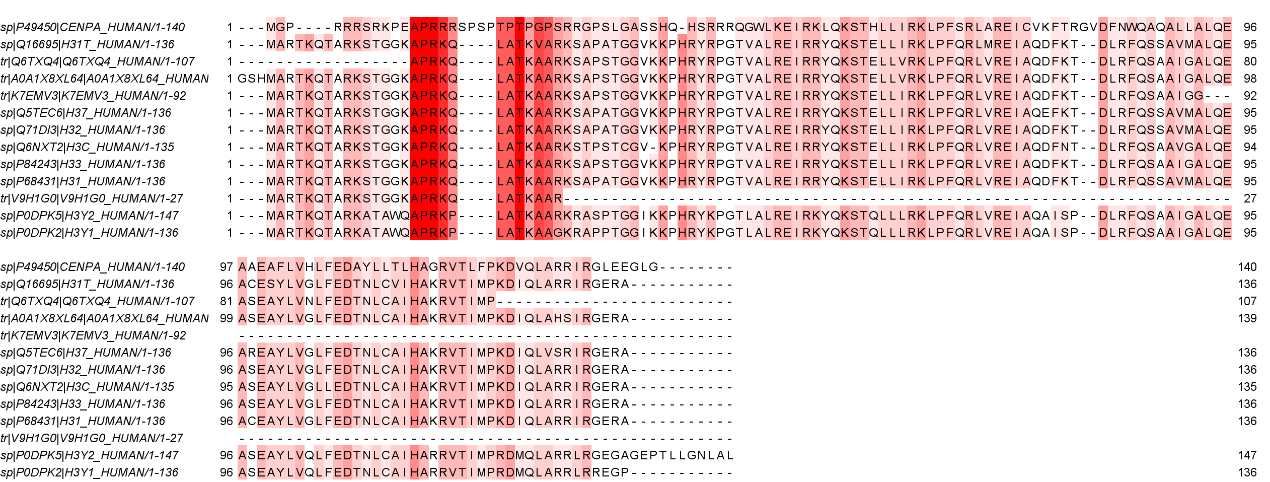


**Supplementary Figure 5.** Multiple sequence alignment of human histone H3 variants in histone interaction network. The sequences are extracted from UniProt^1^ and HistoneDB 2.0^2^ and aligned using Clustal Omega 1.2.4^3^. Conserved sites are shown in red.


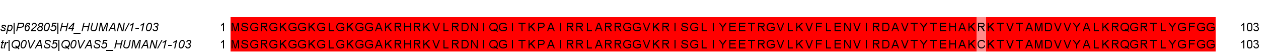


**Supplementary Figure 6.** Multiple sequence alignment of human histone H4 variants in histone interaction network. The sequences are extracted from UniProt^1^ and HistoneDB 2.0^2^ and aligned using Clustal Omega 1.2.4^3^. Conserved sites are shown in red.


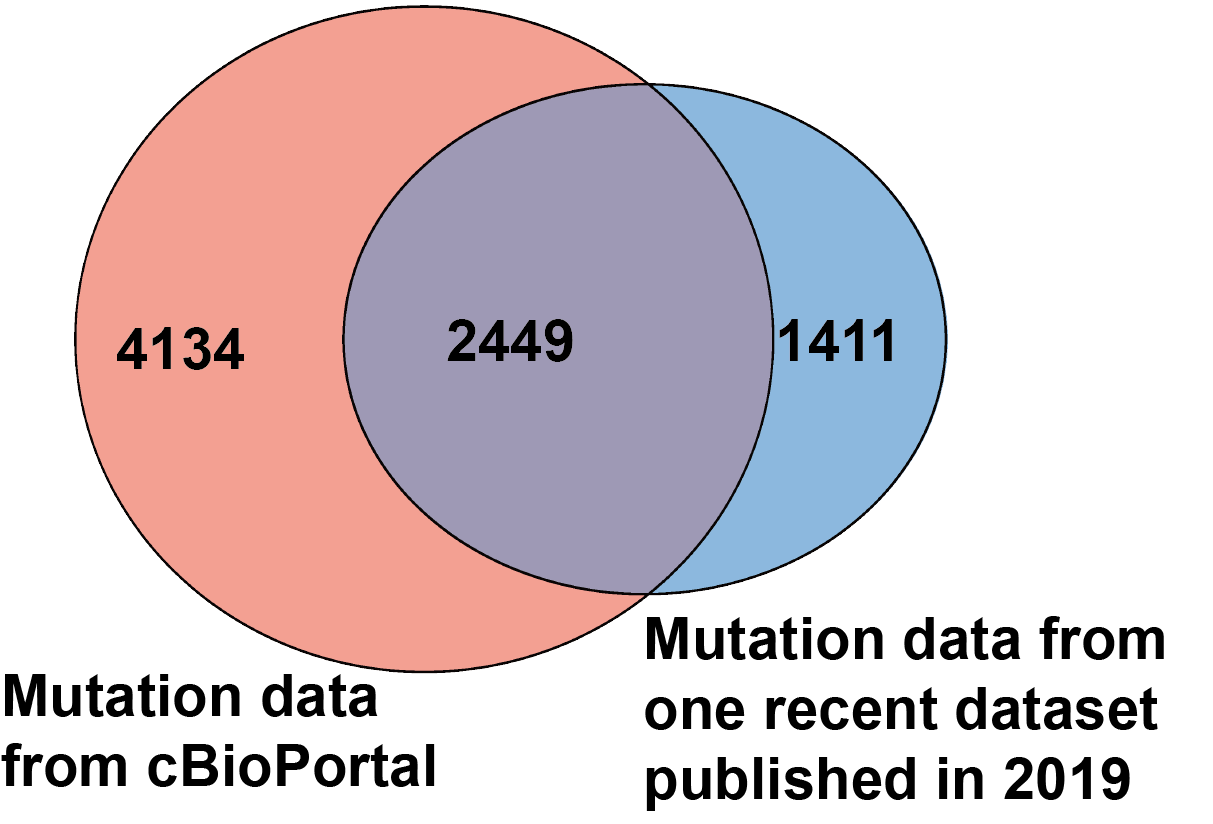


**Supplementary Figure 7.** Comparison of histone cancer mutation data from cBioPortal^4^ and one recent dataset published in 2019^5^.


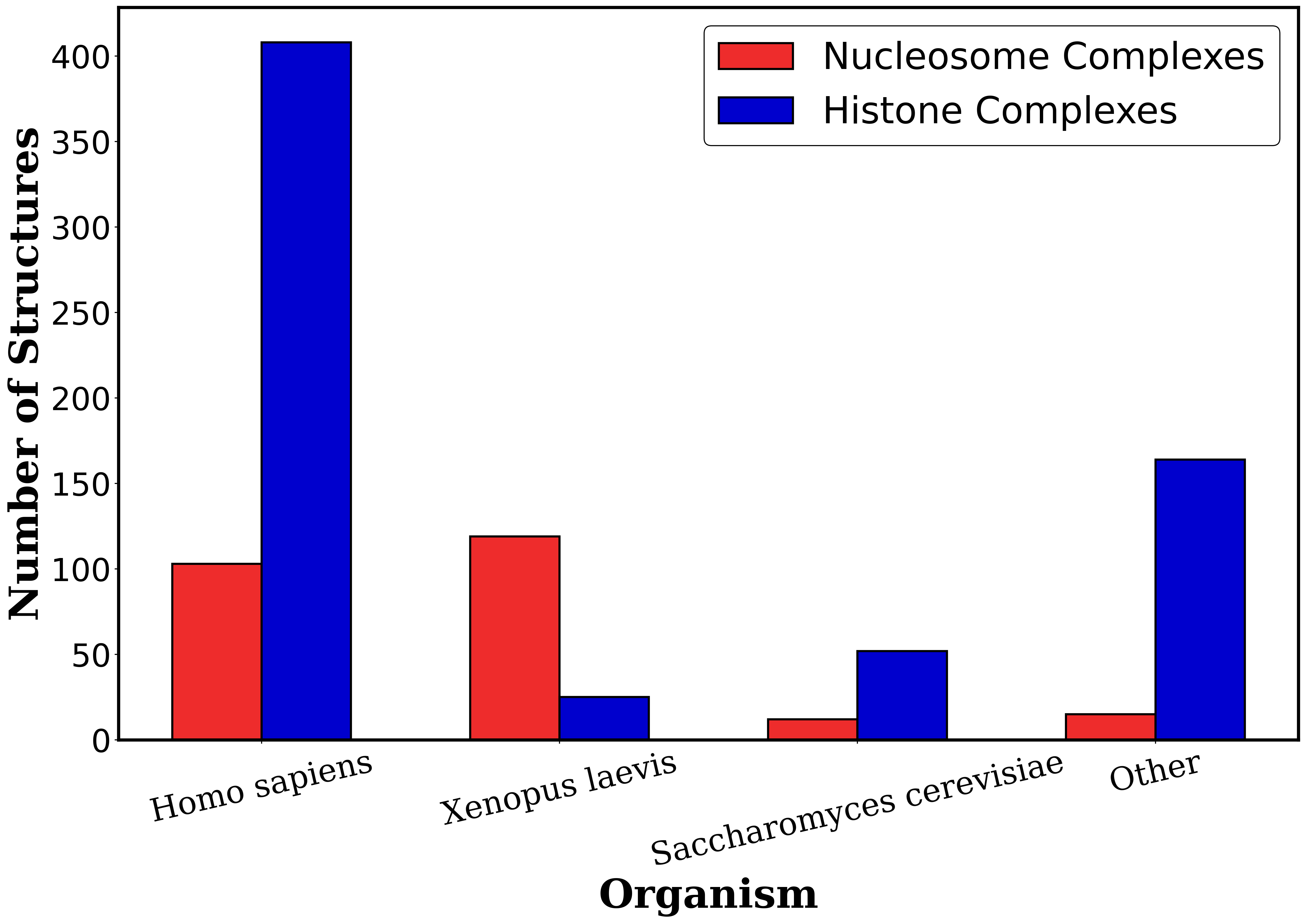


**Supplementary Figure 8.** Number of histone and nucleosome complex structures from different organisms in PDB bank^6^.

**

**

**Supplementary Figure 9.** A degree sorted circle layout is used for visualization of human structural and cross-linking networks at residue-level. Residues of histone H1, H2A, H2B, H3 and H4 are colored as purple, yellow, red, blue and green while residues of histone binding proteins are shown in cyan.


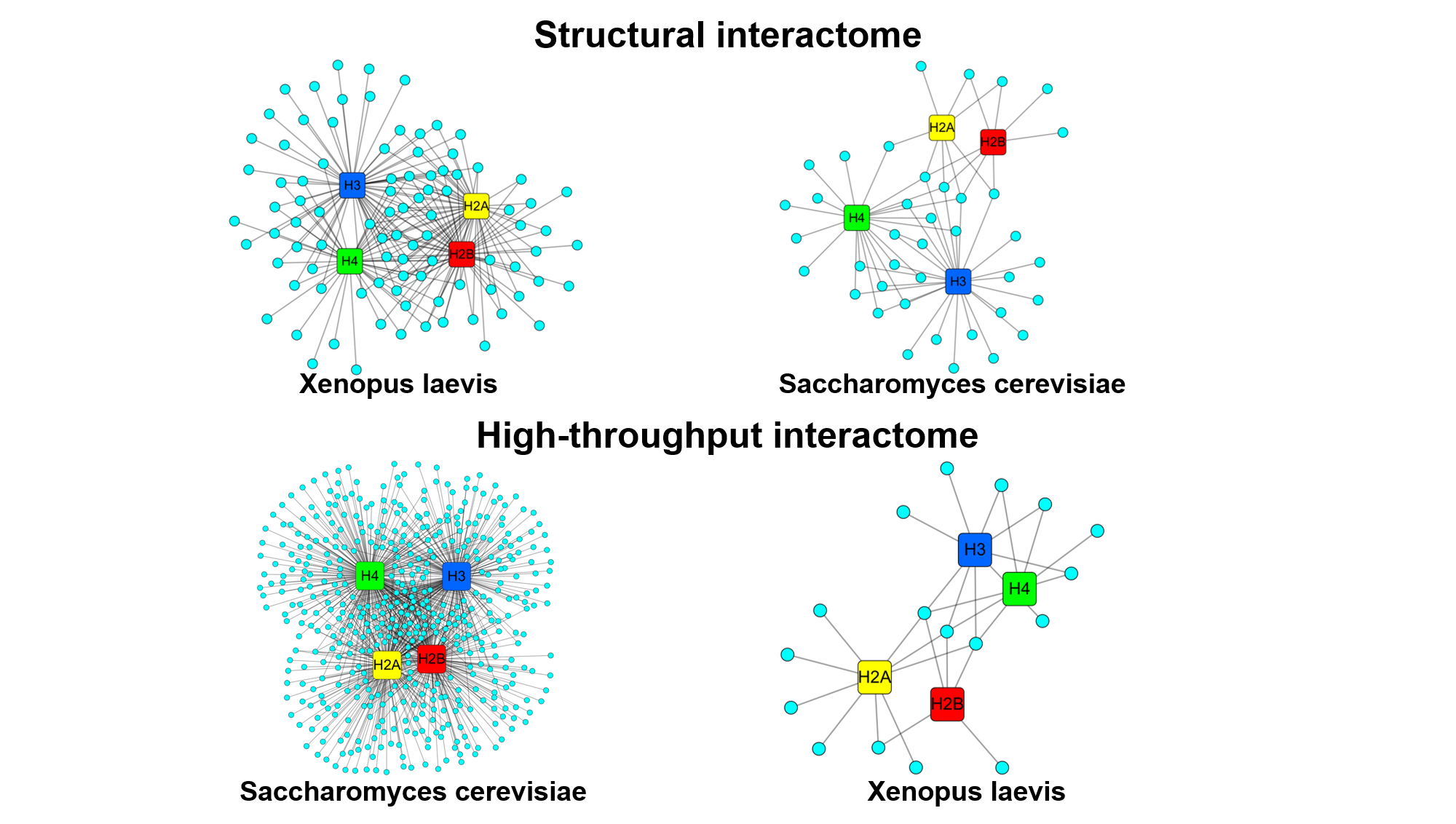


**Supplementary Figure 10.** Structural and high-throughput histone interaction networks at the protein level in Xenopus laevis and Saccharomyces cerevisiae. Histone H2A, H2B, H3 and H4 are colored as yellow, red, blue and green while binding proteins are shown in cyan.


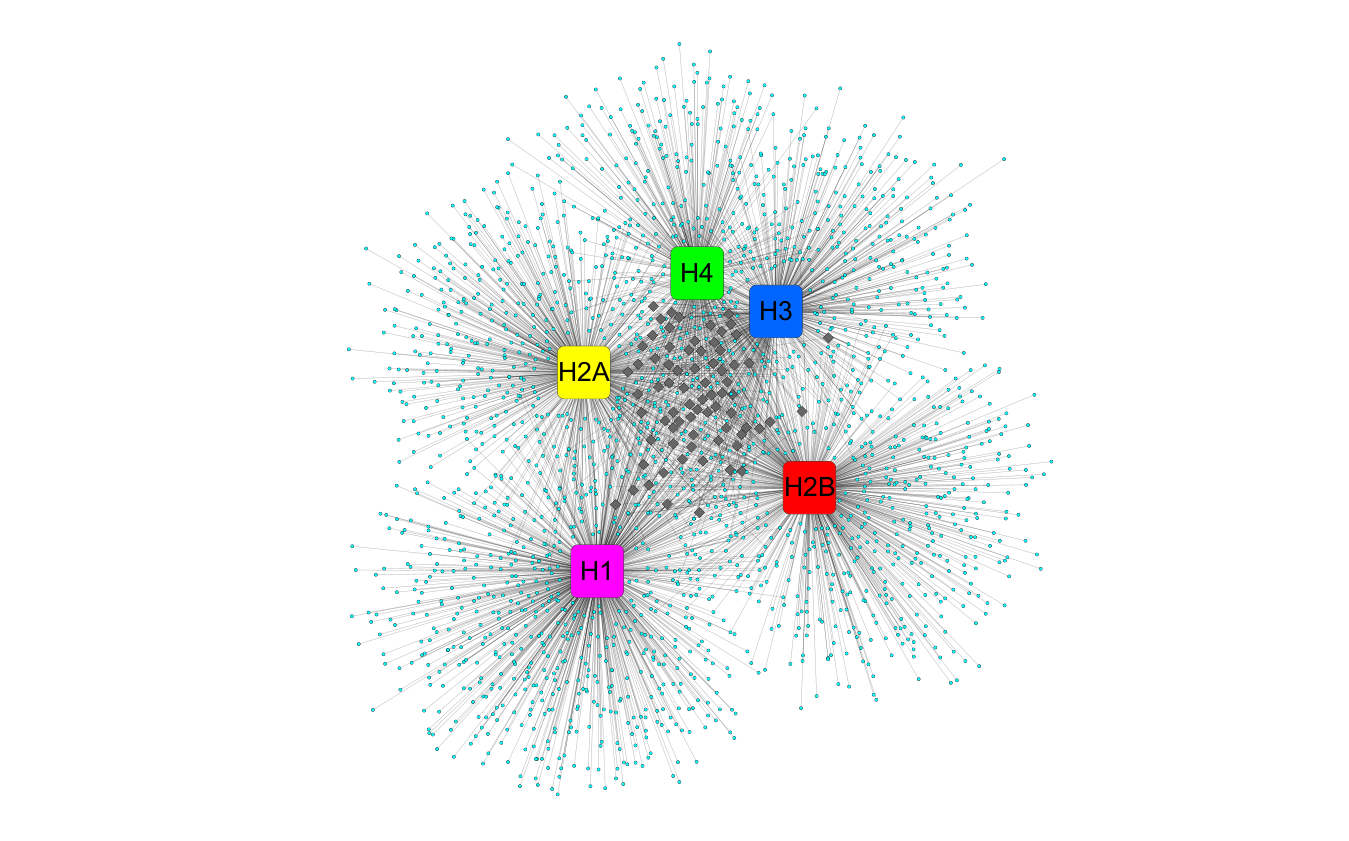


**Supplementary Figure 11.** High-throughput human histone interaction network at the protein level. Histone H1, H2A, H2B, H3 and H4 are colored as purple, yellow, red, blue and green while binding proteins are shown in cyan.

**
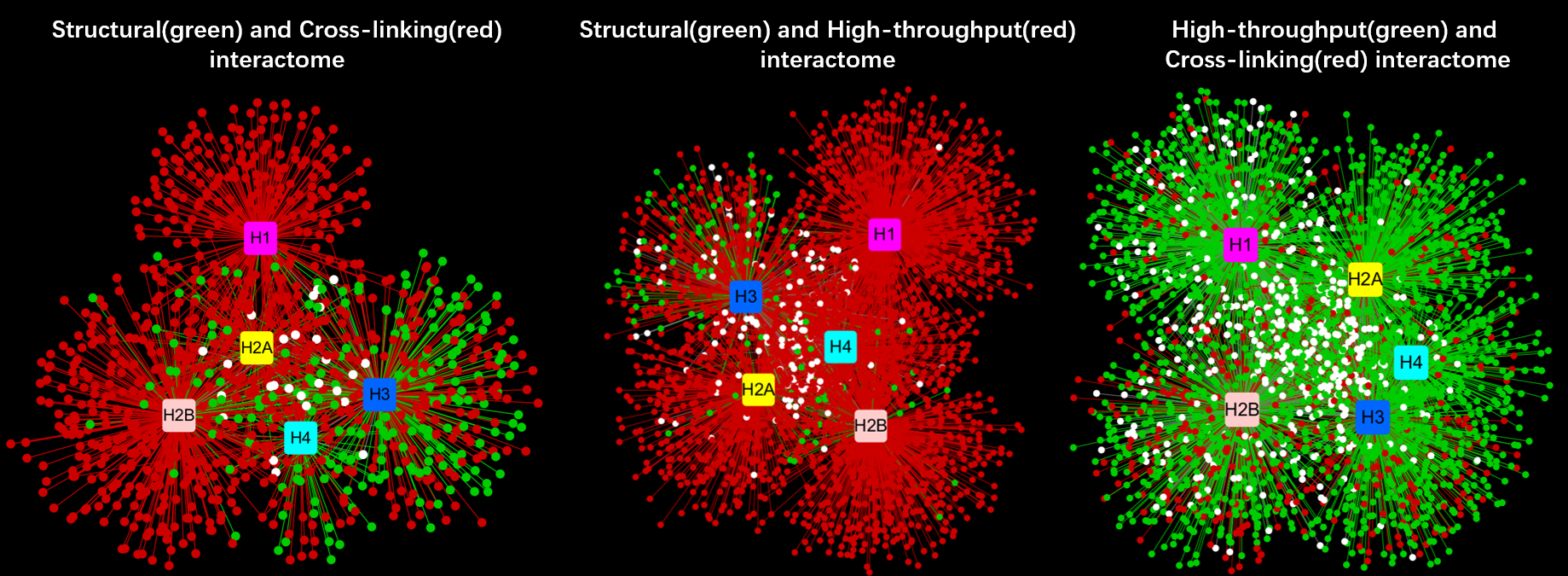
**

**Supplementary Figure 12.** A pair-wise comparison of the human structural, cross-linking and high-throughput interactomes at protein level. Histone H1, H2A, H2B, H3, and H4 are colored as purple, yellow, pink, blue, and cyan. Histone binding proteins from two compared interactomes are colored in red and green respectively, while the shared nodes are shown in white.


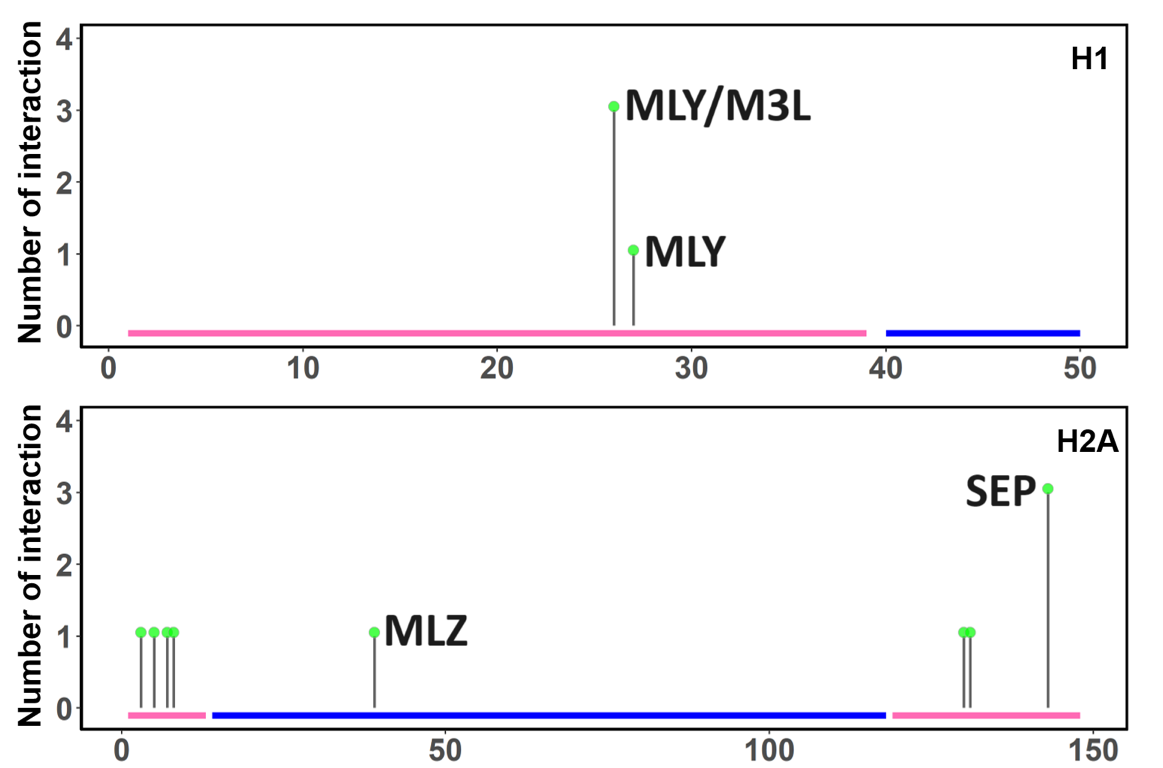


**Supplementary Figure 13.** Number of histone-partner interactions associated with each PTM sites in H1 and H2A. H2B is not shown since no histone-partner interactions associated with histone modifications were identified from available structures. The histone tail and globular regions are indicated by pink and blue bars at bottom. The PTM sites are highlighted with green circles.





**Supplementary Figure 14.** Functional classification of shared histone binding proteins among structural, cross-linking and high-throughput interactomes.


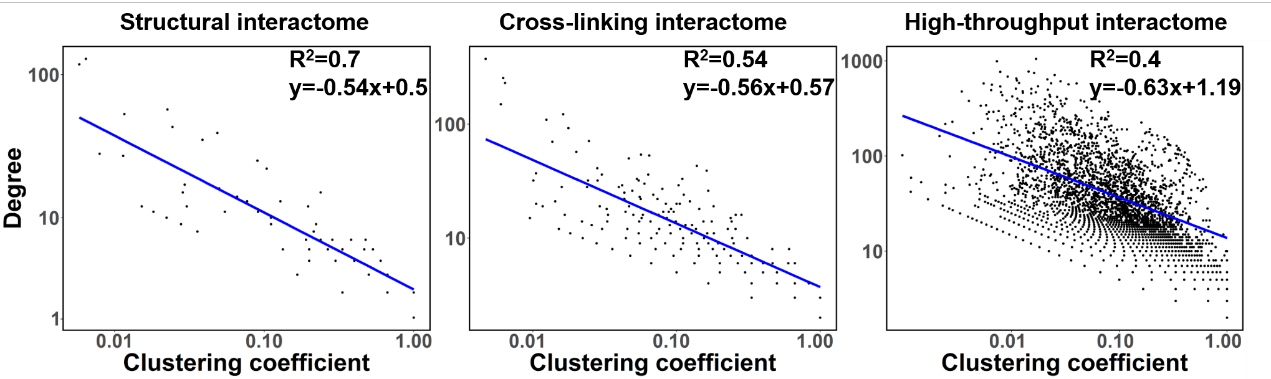


**Supplementary Figure 15.** Dependence of the values of clustering coefficient on node degree in structural, cross-linking and high-throughput histone interactomes.

**
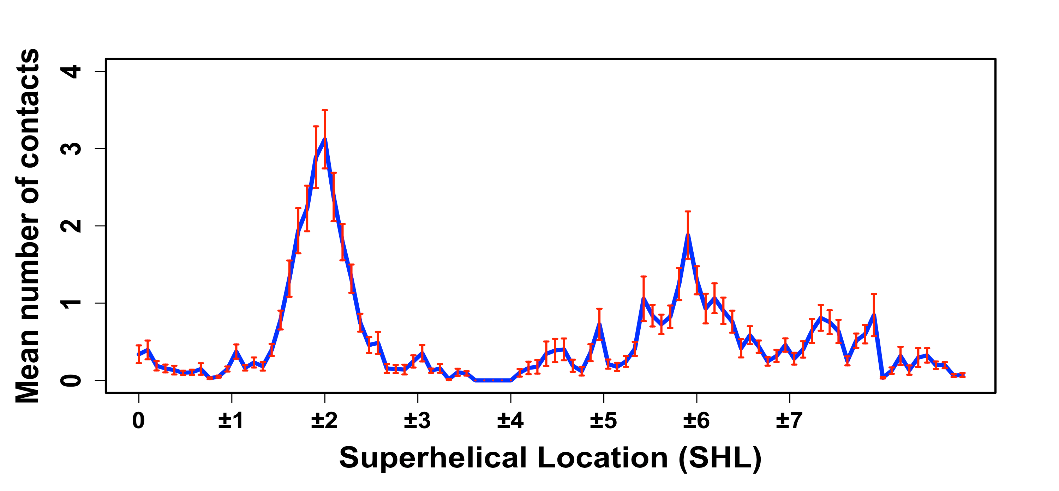
**

**Supplementary Figure 16.** A mean number of contacts between binding proteins and nucleosomal/linker DNA averaged over nucleosome complex structures in the DNA coordinate frame (zero corresponds to the dyad position and super helical locations (SHLs) are shown as integers). The error bars represent standard errors of the mean calculated from different structures.


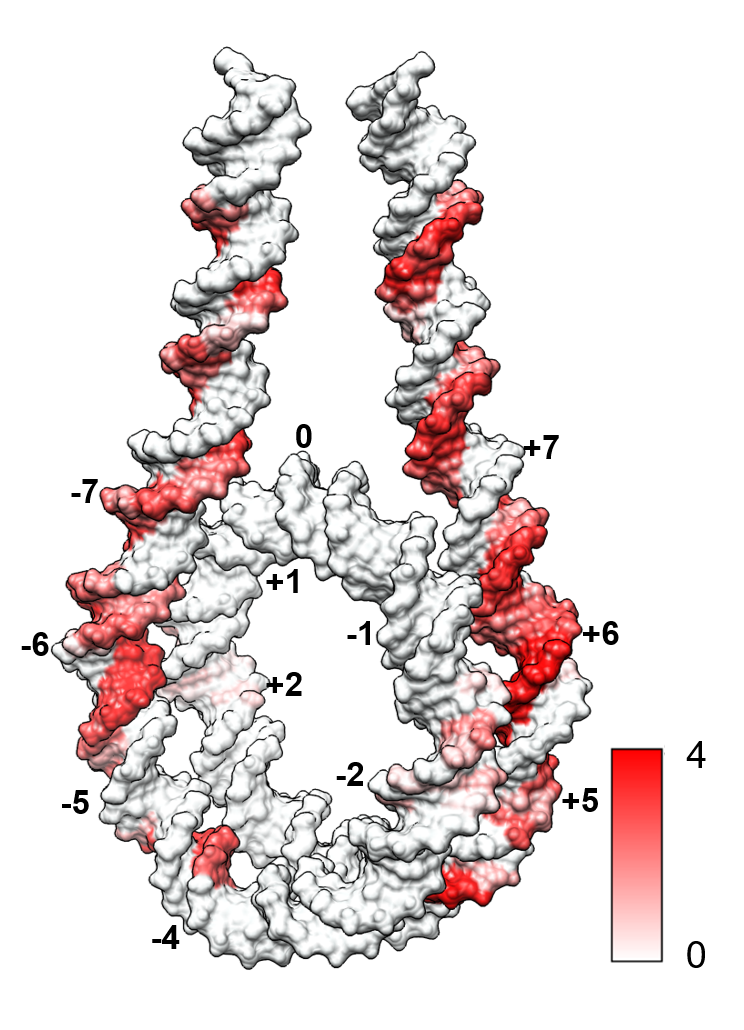


**Supplementary Figure 17.** Mapping of pioneer factor binding sites onto nucleosomal and linker DNA. The number of unique pioneer factor binding per DNA base pairs were mapped onto nucleosomal and linker DNA within nucleosome structure. The DNA representation is generated from PDB 7k5x and histone proteins are not shown.

**
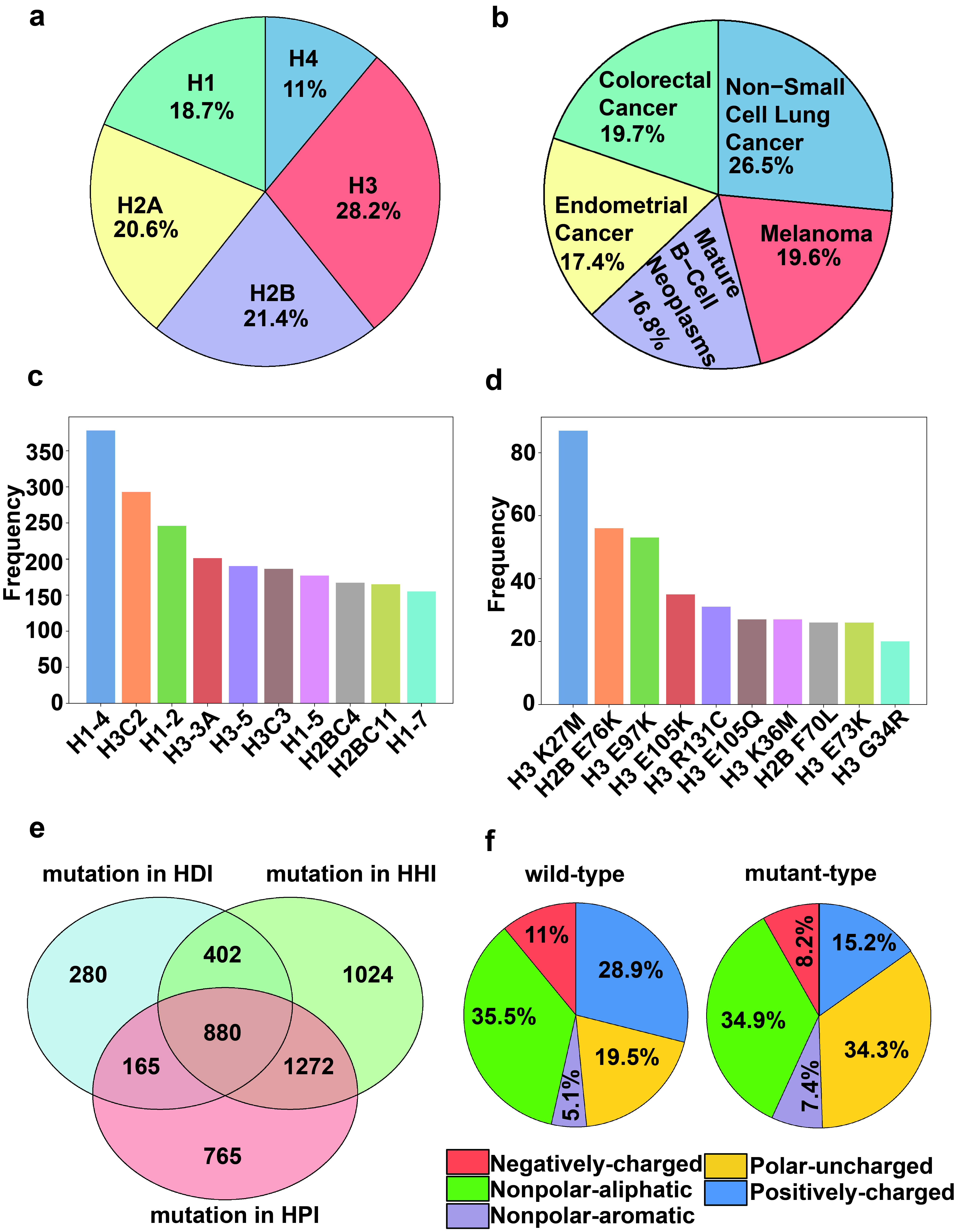
**

**Supplementary Figure 18. Mapping of histone cancer-associated mutations onto human structure histone interaction network.** (a) Percentage of histone cancer-associated mutations per histone type. (b) Percentage of histone cancer-associated mutations per cancer type. (c) Rank of histone genes by their carried number of cancer mutations. (d) Rank of histone cancer mutations by their occurrences. (e) Number of histone cancer mutations mapped onto histone-histone interaction network (HHI), histone-DNA interaction network (HDI) and histone-partner interaction network (HPI). (f) Analysis of physicochemical properties of histone binding interfaces in both wild-type (left) and mutant-type (right). Mutations from the *combined set* were used for the analyses in this Figure.


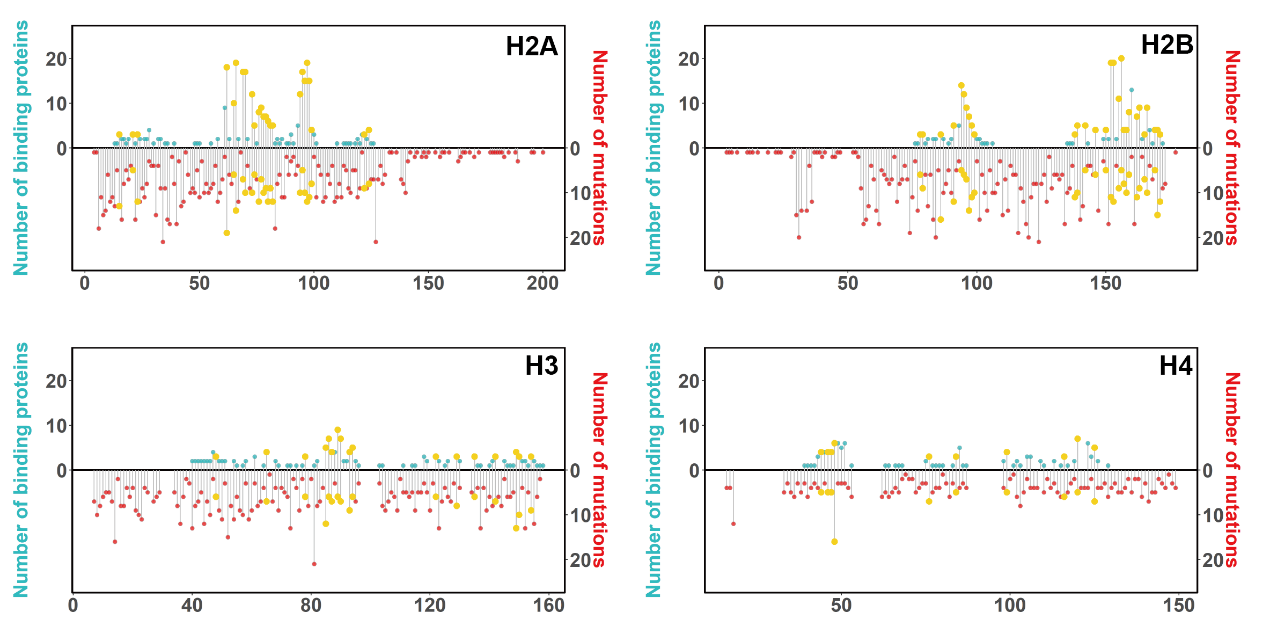


**Supplementary Figure 19.** The number of binding proteins and cancer mutations per residue were mapped onto the consensus sequence of the alignment of histone sequences for each histone type (Supplementary Figure 2-6). Histone residues with at least five mutations and at least three binding proteins at the same time, were highlighted in gold circles.


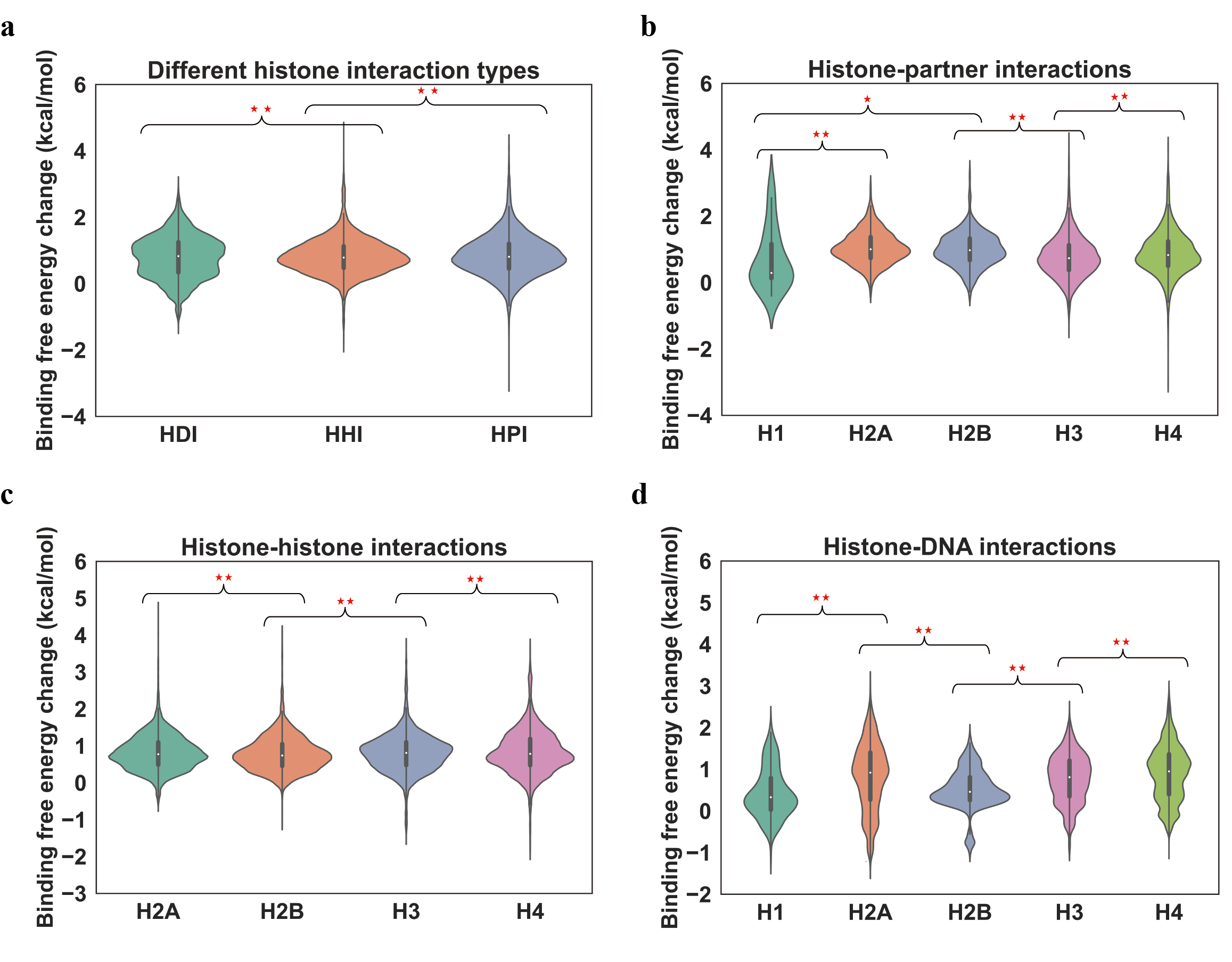


**Supplementary Figure 20.** Analysis of the impact of histone mutations on different types of histone interactions. (a) Distributions of binding free energy changes (∆∆Gs) caused by histone cancer-associated mutations located on different types of histone binding interfaces. (b), (c) and (d) Distributions of ∆∆Gs caused by histone cancer mutations in histone-partner, histone-histone and histone-DNA interactions per histone type. Binding free energy change (∆∆G) values were calculated for each histone mutation that can be mapped onto the human histone structural interaction network. Mutations from the *combined set* were used for the calculation in this Figure. Tukey HSD tests were performed to compare the differences of ∆∆G value distributions and the null hypothesis is that the mean values of ∆∆Gs in two groups are equal. * - p-value < 0.05; ** - p-value < 0.005.


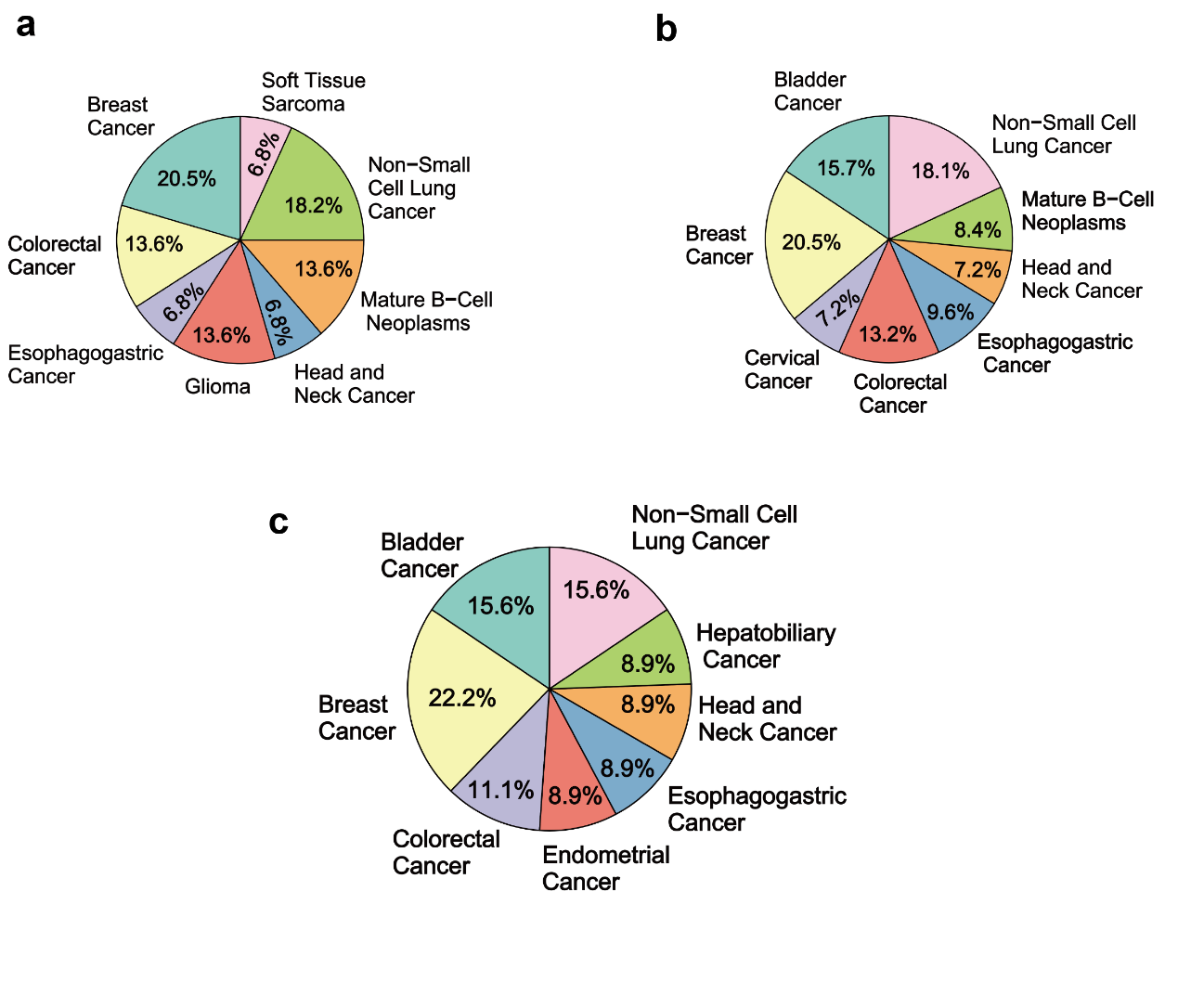


**Supplementary Figure 21.** The most prevalent cancer types for histone cancer mutations predicted to cause largest destabilizing effects on histone-DNA (panel a), histone-histone (panel b) and histone-partner interactions (panel c).

**Supplementary Table 1.** Keywords for the text search of histone/nucleosome structures in PDB.

| Keywords for text search in PDB | histone, CENP, H1, H2A, H2B, H3, H4, H5, nucleosome |
| --- | --- |
| Keywords for histone type identification | H1: H1, histone 1, H5, histone 5  H2A: H2A, histone 2A  H2B: H2B, histone 2B  H3: H3, histone 3  H4: H4, histone 4 |

**Supplementary Table 2.** Number of collected individual nucleosome structures and histone/nucleosome complex structures used for different types of histone interaction mapping.

|  | Experimental method | Number of structures |
| --- | --- | --- |
| Nucleosome complex structure | Electron Microscopy | 218 |
|  | X-ray Diffraction | 16 |
| Histone complex structure | X-ray Diffraction | 596 |
|  | Solution NMR | 46 |
|  | Electron Microscopy | 6 |
|  | Multiple methods | 1 |
| Individual nucleosome structure (without histone binding proteins) | X-ray Diffraction | 178 |
|  | Electron Microscopy | 78 |

**Supplementary Table 3.** Available histone/nucleosome complex structures of different types of organisms in PDB bank^6^ used for mapping histone-partner interactions.

| Organism of histone proteins | Number of histone/nucleosome complex structures |
| --- | --- |
| Homo sapiens | 482 |
| Xenopus laevis | 134 |
| Saccharomyces cerevisiae | 61 |
| Arabidopsis thaliana | 32 |
| Mus musculus | 21 |
| Drosophila melanogaster | 15 |
| Caenorhabditis elegans | 9 |
| Zea mays | 5 |
| Schizosaccharomyces pombe | 4 |
| synthetic construct | 4 |
| Xenopus tropicalis | 4 |
| Gallus gallus | 3 |
| Lama glama | 3 |
| Kluyveromyces lactis | 2 |
| Acropora formosa | 1 |
| Bos taurus | 1 |
| Callosobruchus maculatus | 1 |
| Canis lupus dingo | 1 |
| Drosophila sechellia | 1 |
| Hemicentrotus pulcherrimus | 1 |
| Latimeria chalumnae | 1 |
| Nicotiana tabacum | 1 |
| Ophiophagus hannah | 1 |
| Schizosaccharomyces japonicus | 1 |
| Siphlonella sp. BYU IGCEP083 | 1 |
| Strongylocentrotus purpuratus | 1 |
| Urechis caupo | 1 |
| Odobenus rosmarus divergens | 1 |
| Camelus ferus | 1 |
| Fundulus heteroclitus | 1 |

**Supplementary Table 4.** Summary of constructed human histone interactomes at three levels of granularities (protein-level, domain-level and residue-level).

| Interactome type | Level | number of edges | number of nodes | number of structures |
| --- | --- | --- | --- | --- |
| Structural (histone-partner interaction) | protein | 327 | 220 | 482 |
|  | domain | 195 | 152 | 388 |
|  | residue | 14,498 | 6,118 | 482 |
| Cross-linking | protein | 1,046 | 754 | NA |
|  | domain | 464 | 306 | NA |
|  | residue | 2,088 | 1,267 | NA |
| High-throughput | protein | 2,963 | 2,103 | NA |
| Combined interaction network (structural and cross-linking) | protein | 1,355 | 943 | NA |

**Supplementary Table 5.** Summary of constructed human histone global interactomes by adding one additional layer of proteins that interact with histone-binding partners.

| Type | Level | Number of edges | Number of nodes | Number of structures |
| --- | --- | --- | --- | --- |
| Structural interactome | protein | 1,100 | 783 | 2232 |
| Cross-linking interactome | protein | 3,598 | 1,786 | NA |
| High-throughput interactome | protein | 118,947 | 17,949 | NA |
| Combined interactome (structural and cross-linking) | protein | 4,688 | 2,465 | NA |
| Combined interactome (structural, cross-linking and high-throughput) | protein | 122,638 | 18,298 | NA |

**Supplementary Table 6**. 83 representative nucleosome complex structures used for nucleosome-partner binding mode analyses.

| PDB ID | 3TU4, 6FTX, 7UV9, 7VVU, 7ZS9, 6T7B, 5KGF, 6G0L, 7Y7I, 8G8G, 7ZSA, 7EG6, 7ENN, 6RYU, 5GTC, 6YOV, 6TDA, 7EGP, 7U50, 6VEN, 6S01, 6T90, 6KW3, 6GEJ, 6LTJ, 6PA7, 7PH6, 7E9F, 6T9L, 7OHA, 8H1T, 7XCT, 6VYP, 7XZZ, 6E0P, 7OH9, 7LYC, 7BWD, 6R1U, 7Y8R, 6NZO, 7XD0, 7LYB, 6Z6P, 6R25, 5MLU, 7D1Z, 6R8Z, 7SCZ, 7W9V, 8GPN, 8GRM, 7EA8, 6R91, 7SSA, 5E5A, 6KIU, 6WKR, 8F86, 6R90, 6QLD, 8AV6, 6PWV, 6PWX, 5X0X, 7TAN, 7YWX, 8ATF, 6KIW, 8DU4, 5X0Y, 6USJ, 6MUP, 6PWF, 7E8D, 3MVD, 6NE3, 6X0N, 7CCQ, 6JYL, 7CRQ, 8H0V, 6T7C |
| --- | --- |

**Supplementary Table 7**. Classification of 83 representative structures using the functional annotations from NucleosomeDB^7^ and Uniprot^1^.

| Function of complexes | Number of structures | PDB ID |
| --- | --- | --- |
| PTM writers | 23 | 6KIU, 6KIW, 6NZO, 6PWV, 6PWX, 6USJ, 6VEN, 6WKR, 6X0N, 7BWD, 7CRQ, 7D1Z, 7E8D, 7EA8, 7LYB, 7LYC, 7SCZ, 7TAN, 7VVU, 7W9V, 7XCT, 8DU4, 8GRM |
| Chromatin remodelers | 18 | 5X0X, 5X0Y, 6FTX, 6LTJ, 6G0L, 6GEJ, 6JYL, 6KW3, 8ATF, 6PWF, 6RYU, 6TDA, 7EG6, 7EGP, 7ENN, 7Y8R, 8ATF, 8AV6, 6NE3 |
| Transcription factors | 11 | 6S01, 6T7B, 6T7C, 6T90, 6YOV, 7OH9, 7OHA, 7PH6, 7SSA, 7XZZ, 8G8G |
| PTM erasers | 8 | 6R1U, 6R25, 6T9L, 6VYP, 6Z6P, 7UV9, 8F86, 8H1T |
| PTM readers | 7 | 5KGF, 8GPN, 6FTX, 6R1U, 6R25, 6S01, 6VEN |
| Pioneer factors | 7 | 6T7B, 6T7C, 6T90, 6YOV, 7SSA, 7XZZ, 8G8G |
| DNA repair | 5 | 5KGF, 6R8Z, 6R90, 6R91, 7U50 |
| Kinetochore components | 4 | 6E0P, 6MUP, 6QLD, 7YWX |
| RNA polymerases | 3 | 7ZS9, 7ZSA, 8H0V |
| Viral gene regulation | 2 | 5E5A, 5GTC |
| RanGTP gradient signal | 1 | 3MVD |
| Transcriptional silencing | 1 | 3TU4 |
| Centromere formation | 1 | 7Y7I |
| cGAS | 1 | 7CCQ |
| DNA integrations components | 1 | 5MLU |
| DNA methyl-transferases | 1 | 6PA7 |
| DNA replication | 1 | 7E9F |
| Ubiquitination | 1 | 7XD0 |
| Histone exchange | 1 | 6GEJ |
| Meiosis | 1 | 7E9F |

**Supplementary Table 8**. Histone genes used for collecting histone cancer mutations from cBioportal^8^.

| Histone type | Histone gene |
| --- | --- |
| H1 | H1-0, H1-1, H1-2, H1-3, H1-4, H1-5, H1-6, H1-7, H1-8, H1-8, H1-9P, H1-10, H1-12P |
| H2A | H2AC1, H2AC2P, H2AC3P, H2AC4, H2AC5P, H2AC6, H2AC7, H2AC8, H2AC9P, H2AC10P, H2AC11, H2AC12, H2AC13, H2AC14, H2AC15, H2AC16, H2AC17, H2AC18, H2AC19, H2AC20, H2AC21, H2AC25, H2AZ1, H2AZ1, H2AZ2, H2AZ2, H2AZ2, H2AZ2, H2AZ2, MACROH2A1, MACROH2A1, MACROH2A1, MACROH2A2, H2AX, H2AJ, H2AB1, H2AB2, H2AB3, H2AP, H2AQ1P, H2AL1Q, H2AL1MP, H2AL3 |
| H2B | H2BC1, H2BC2P, H2BC3, H2BC4, H2BC5, H2BC6, H2BC7, H2BC8, H2BC9, H2BC10, H2BC11, H2BC12, H2BC13, H2BC14, H2BC15, H2BC16P, H2BC17, H2BC18, H2BC18, H2BC19P, H2BC20P, H2BC21, H2BC26, H2BC27P, H2BK1, H2BL1P, H2BW1, H2BW2, H2BW3P, H2BW4P, H2BN1, H2BC12L |
| H3 | H3C1, H3C2, H3C3, H3C4, H3C5P, H3C6, H3C7, H3C8, H3C9P, H3C10, H3C11, H3C12, H3C13, H3C14, H3C15, H3-3A, H3-3B, H3-4, H3-5, H3P16, H3-7, H3P44, H3Y1, H3Y2, CENPA, CENPA |
| H4 | H4C1, H4C2, H4C3, H4C4, H4C5, H4C6, H4C7, H4C8, H4C9, H4C10P, H4C11, H4C12, H4C13, H4C14, H4C15, H4C16 |

**Supplementary Table 9.** Identifications of hub nodes (MCC≥4) in human histone structural, cross**-**linking and combined interactomes at protein level using MCC (Maximal Clique Centrality) scores.

| Interactome type | Hub nodes |
| --- | --- |
| Structural | Q92560, C4QYQ8, Q99728, Q8TEK3, Q03188, G0S589, Q96H22, P0CG47, Q9Y5B9, Q9NXA8, P0AEX9, Q12888, Q9NQR1 |
| Cross-linking | P07814, O15417, P12236, Q8WZ42, P17096, Q03164, P05204, P62987, P62979, O95425, Q68DE3, P48741, Q09666, Q03001, P04406, Q9Y673, Q14683, Q32P51, Q9Y3U8, P02545, Q8WUA2, O43615, Q00839, P22626, Q53GA4, P20929, P62826, P62424, O00268, Q9BR39, P05114 |
| Combined  (structural and cross-linking) | Q03164, P62979, P62987, P05204, P17096, Q8WZ42, P12236, O15417, P07814, Q9NQR1, Q12888, P46100, P55201, P0AEX9, Q9NXA8, O60885, Q9Y5B9, P0CG47, Q96H22, G0S589, Q03188, Q8TEK3, Q99728, C4QYQ8, Q92560, P05114, Q9BR39, O00268, P62424, P62826, P20929, Q53GA4, P22626, Q00839, O43615, Q8WUA2, P02545, Q9Y3U8, Q32P51, Q14683, Q9Y673, P04406, Q03001, Q09666, P48741, Q68DE3, O95425 |

**Supplementary Table 10.** Identifications of hub nodes (MCC≥4) in human histone structural, cross**-**linking and combined interactomes at domain level using MCC (Maximal Clique Centrality) scores.

| Interactome type | Hub nodes |
| --- | --- |
| Structural | SIRT5_Af1_CobB, Ubl_ubiquitin, CENP-N, DOT1, CENP-C_mid |
| Cross-linking | Ubl_ubiquitin, HMG14_17, Ig_Titin_like, WEPRS_RNA,  WHEP-TRS, Ribosomal_L40e, TAF4, RAN, RRM2_hnRNPA2B1, Myosin_tail_1, 3a0801s03tim44, Filament, Ribosomal_L36e, SMC_N, DPG_synthase, CH_DYST_rpt1, NEBU, RRM1_hnRNPA1, bHLHzip_USF3 |
| Combined  (structural and cross-linking) | Ubl_ubiquitin, HMG14_17, Ig_Titin_like, WEPRS_RNA,  WHEP-TRS, Ribosomal_L40e, SIRT5_Af1_CobB, Bromo_Brdt_II_like, CENP-N, RNAP_II_RPB1_N, DOT1,  CENP-C_mid, TAF4, RAN, RRM2_hnRNPA2B1, Myosin_tail_1, 3a0801s03tim44, Filament, Ribosomal_L36e, SMC_N, DPG_synthase, CH_DYST_rpt1, NEBU, RRM1_hnRNPA1, bHLHzip_USF3 |

**Supplementary Table 11.** Number of identified histone/nucleosome-partner interactions per organism.

| Type of organism for histone proteins | Number of histone-partner interactions  at protein level | Number of histone/nucleosome complex structures |
| --- | --- | --- |
| Human | 327 | 482 |
| Xenopus laevis | 203 | 134 |
| Saccharomyces cerevisiae | 63 | 61 |
| Other | 134 | 175 |

**Supplementary Table 12.** Number of identified histone interactions per interaction type.

| Type of histone interaction | Number of  interactions at protein level | Number of used histone/nucleosome structures |
| --- | --- | --- |
| Histone-partner | 627 | 842 |
| Histone-histone | 339 | 568 |
| Histone-DNA | 100 | 490 |
| DNA-partner | 142 | 202 |

**Supplementary Table 13.** List of histone variants included in histone-histone and histone-DNA interaction network.

| Histone type | Uniprot ID: Histone protein name |
| --- | --- |
| H1 | p07305: Histone H1.0, p02259: **Histone H5**  p22844: **Histone H1.0-B,** q92522: Histone H1.10  p10412: Histone H1.4, p16403: Histone H1.2 |
| H2A | p06897: Histone H2A type, p02263: Histone H2A-IV  p0c0s5: Histone H2A.Z, p04911: Histone H2A.1  o75367: Core histone macro-H2A.1, p04908: Histone H2A type 1-B/E  q8cgp6: Histone H2A type 1-H, p84051: Histone H2A  q6azj8: Histone H2A, q71ui9: Histone H2A.V  q8cgp4: Histone H2A, p04912: Histone H2A.2  c0hke1: Histone H2A type 1-B, q96qv6: Histone H2A type 1-A  p20671: Histone H2A type 1-D, p0c0s8: Histone H2A type 1  q93077: Histone H2A type 1-C, p16104: Histone H2AX  q9btm1: Histone H2A.J, o81826: Probable histone H2A.3  p0c5z0: Histone H2A-Bbd type 2/3, p0ch09: Histone H2A type 1  q6fi13: Histone H2A type 2-A, q6ck59: Histone H2A  q16777: Histone H2A type 2-C, q9ld28: Histone H2A.6  a2x254: Core histone macro-H2A.1, a8bhs7: Histone H2A  q6dke3: Histone H2A, a0a097i2b5: Histone doublet H2B-H2A  d2xb49: Histone H2B/H2A fusion protein, p0c0s6: Histone H2A.Z  b2r5b3: Histone H2A, a0a1b2jd99: Histone H2A  a0a8c0k5d3: Histone H2A |
| H2B | p02281: Histone H2B 1.1, p0c1h5: Histone H2B 7  p02294: Histone H2B.2, q9d2u9: H2B.U histone 2  p0c1h3: Histone H2B 1/2/3/4/6, o60814: Histone H2B type 1-K  p02283: Histone H2B, p06899: Histone H2B type 1-J  p0c1h4: Histone H2B 5, q28d68: Histone H2B  q96a08: Histone H2B type 1-A, p33778: Histone H2B type 1-B  p70696: Histone H2B type 1-A, g0sdn1: Histone H2B 1.1  p02293: Histone H2B.1, p62807: Histone H2B type 1-C/E/F/G/I  q16778: Histone H2B type 2-E, q9lqq4: Histone H2B.1  q5qnw6: Histone H2B type 2-F, q6ck60: Histone H2B.1  b4dr52: Histone H2B, a8bi78: Histone H2B  a0a1b2jbs1: Histone H2B, q2m2t1: Histone H2B type 1-K  s9wx78: Histone H2B, a0a8j0u496: Histone H2B |
| H3 | p02302: Histone H3.3C, p84229: Histone H3.2  p84233: Histone H3.2, p61830: Histone H3  p68433: Histone H3.1, p68431: Histone H3.1  p02299: Histone H3, q6cti2: Histone H3-like centromeric protein CSE4  q16695: Histone H3.1t, p49450: Histone H3-like centromeric protein A  q71di3: Histone H3.2, p84243: Histone H3.3  q92133: Histone H3, q6nxt2: Histone H3.3C  q6pi79: Histone H3.3, p0dpk2: Histone H3.Y  a0a1x8xl64: Histone H3, p84244: Histone H3.3  s4raz3: H3.4 histone, a0a310ttq1: Histone H3  q4qhb5: Histone H3, p36012: Histone H3-like centromeric protein CSE4  q9v6q2: Histone H3-like centromeric protein cid, e2ru29: Histone H3  q6xxm1: Histone H3-like centromeric protein A, d2xb48: Histone H3  a0a097i2d0: Histone doublet H4-H3, a0a1b2jb78: Histone H3  a0a2u3zmz6: Histone H3, a0a6i9khi6: Histone H3  a0a653dhj5: Histone H3 |
| H4 | p62799: Histone H4, p62801: Histone H4  p62806: Histone H4, p02309: Histone H4  p62805: Histone H4, p84040: Histone H4  q6cmu6: Histone H4, a8buj9: Histone H4  a0a1b2ja70: Histone H4, h3a5h1: Histone H4  a0a3q2ss86: Histone H4, a0a0p9axl3: Histone H4 |

**Supplementary Table 14.** Histone binding proteins shared between structural, cross-linking and high-throughput interactomes.

|  | UniProt IDs |
| --- | --- |
| Histone binding proteins shared in structural and cross-linking interactomes | P63104, Q5VWG9, Q13185, P62979, P49736, O96028, Q14839, P55197, O60885, P26358, P61077, P09874, P46100, Q8NEZ4, Q9BTC0, Q14974, P45973, Q15059, P55201, P23497, Q9Y5B9, Q08945, Q03164, Q15910, Q7Z4V5, O15054 |
| Histone binding proteins shared in structural and high-throughput interactomes | Q8WTS6, P17693, Q9NQR1, Q14676, O14646, P63104, P61964, Q12830, Q12888, Q9Y294, Q92784, Q14839, P46100, Q96T88, O43189, P42568, O75164, Q96BD5, Q9Y468, Q92793, Q9H0M4, P55201, O60341, Q9UNL4,  Q16576, P45973, O43791, O75530, O75151, Q9UPP1, Q03164, O15164, Q7Z4V5, Q99549, Q8NCD3, Q8NEM0, Q6ZW49, Q13185, O60885, Q92794, Q8N6T7, Q5T6S3, Q9BTT0, Q9UQR0, Q8NB78, Q9BZ95, O14744, Q49A26, Q9UER7, Q6B0I6, Q9H9B1, O94953, Q582G4, Q8R5C8, Q8TF76, Q9Y6K1, Q7JXA8, Q86UY6, Q8IXJ6, P21675, Q9Y5B9, P49736, Q9NVP2, Q9NTG7, Q5VWG9, P55197, Q86X55, Q8NEZ4, P23497, Q15906, Q9QR71, Q15059, Q15910, Q03111, Q96HA7, Q96KQ7, P0CG47, O15054, P83916, Q9UIF9, Q9BVI0, Q8WYB5, P62979, P26358, Q9Z1B8, Q9Y483, O95619, C4QZQ7, Q15047, Q96H22, Q9ULG1, P35189, Q03188, Q14974, O14686, Q92466, Q08945, O14929, Q8WWQ0, Q8TEK3, Q8WXX5, Q9ULU4, O96028, P38398, Q99728, P09874, Q99986, Q9Y2K7, P49321, P30117, Q09472, Q12824, Q9UNP9, Q99496, Q92560, P61077, P25554 |
| Histone binding proteins shared in cross-linking and high-throughput interactomes | P62979, P62987, P05204, P24928, P05114, Q9BR39, O00268, Q03164, P0DMV8, P62424, Q8TAQ2, P62826, P20929, Q8WTT2, P51531, Q9H7Z7, P17096, P35268, Q9H7N4, Q8WZ42, Q8IYB3, Q9Y4B4, P61247, P47914, P22626, P09651, P48634, P12236, Q9UQE7, Q9Y520, P82930, O94906, P49736, Q2NL82, O15417, Q9C0D5, O95922, P55209, Q9UKX7, P61513, P62851, P06748, P46087, Q9NTI5, Q00839, P62841, P46821, Q9H0A0, Q13813, Q8WUA2, P61326, P02545, Q53EL6, P22415, P25490, P84098, Q86VM9, Q8NB66, P62753, Q96SU4, Q13459, Q8N3U4, Q8NEZ4, O95239, P19338, P26583, Q76FK4, P05455, P23396, Q9Y5T5, Q08945, P62316, Q13151, Q9Y230, Q14980, A8MW92, Q14690, P36873, P11717, P11388, O43395, Q9NQG5, P23497, P36578, P50914, P24043, O94826, P35579, Q02878, P63279, P46013, P61978, O15042, Q15003, Q99729, O15056, Q9Y2W1, P46783, O60287, O75116, A3KN83, Q9GZR2, Q9NR22, Q9Y265, O43390, O60506, Q96LB3, P62750, P62910, Q5VWG9, P83731, Q9Y2S6, P49591, Q5JSZ5, Q8N567, P30519, Q14974, Q14696, Q14566, Q9Y2K3, O75475, Q9Y3B7, Q14683, P10586, Q8NF91, Q9P2E9, P27816, Q96N64, P60866, Q6ZQQ6, P13667, P40926, A6NHR9, P00558, A4UGR9, Q92576, O95819, P22694, P30101, Q99575, O60264, P52926, Q01105, Q92954, Q9UM54, Q8WX93, P46781, P12956, Q15637, O96028, Q00987, O60673, Q96QH2, Q15651, Q5SSJ5, P61077, Q9Y6X0, Q16666, P33991, P63104, P09429, Q6PKG0, P27348, P10636, Q9H7B2, Q15397, Q5JTH9, O15355, P07910, O15347, Q9Y5B9, Q96ME7, P07814, P04406, P06733, Q9P225, Q9P2D7, P60842, O15078, Q99848, Q8IVF4, Q8WY36, Q8IWX8, Q01484, P46100, Q07065, Q16658, P15924, Q15910, Q15029, Q9UIG0, A1A4S6, O60832, P35659, P68104, P00533, Q9NR30, Q09666, Q03701, O95433, P20020, Q16531, Q9NY12, Q96GQ7, P26641, P51114, P78347, Q14839, Q5W0A0, Q13315, P51858, Q14004, Q86WP2, P55201, P38919, P49454, Q96CT7, Q8N5Q1, O60885, Q15059, Q13561, Q96QE3, Q15398, Q8NE71, Q9BXW9, Q92785, P47756, Q13185, P54132, Q86VP6, Q6V0I7, Q9BWV3, P01023, Q12955, Q9UKJ3, Q9GZR7, Q7Z4V5, Q96CS3, O94923, Q7Z4H7, P48741, Q14512, O00160, O95084, Q9NQZ2, P33992, Q9H6T0, P16471, O75521, Q7Z7G8, P23560, Q14678, Q8IY18, Q86XJ1, Q9NVQ4, Q5VZL5, Q13426, Q9P1P4, O14958, Q8IUX8, P26196, Q6IN85, P35499, P82914, Q68DD2, O75912, Q14642, O15090, Q8WYK1, Q9Y236, Q9NQ66, P12036, Q6IC83, Q8TD16, Q7Z6B0, O94810, Q8NDI1, Q9H892, O15054, Q9UJT2, Q6ZN66, Q8N6M6, O43295, Q07092, Q8WUU4, O43586, Q9Y6Q1, Q68DE3, Q9UHW9, Q9Y4K4, Q8N6H7, P18858, P14625, Q8WYJ6, O94933, P29120, Q8NEN9, Q6NXP0, P35269, Q13956, Q9BYN7, Q86Y22, Q8IYW2, Q96MN5, Q3YEC7, Q6PIW4, O75844, Q9Y4L1, Q5JY77, A6NJL1, Q8N4N8, Q9HCK1, P49641, P35626, Q7L2E3, Q99626, Q9BQI3, Q96EZ8, Q8IYX3, O95425, P55197, Q9UHB7, P18754, P62861, O94874, Q13127, Q5T0F9, Q14764, Q5HYL7, P52756, Q9UPP5, Q8NHP7, Q9Y5S2, Q96MT7, Q5VUJ6, P09874, Q8TE59, P26358, Q5KSL6, Q96JG9, B1AJZ9, Q96MG2, P59046, Q96KJ9, Q86YN1, Q9Y251, Q9UKX2, Q5BKZ1, Q9BW71, Q8IWU5, P52948, Q9BUD6, Q13889, P43155, Q496A3, Q15554, Q96LI9, Q5F1R6, P48995, Q6P2Q9, Q96D70, P98171, Q70EL2, Q3SY52, Q15843, O75054, Q15361, Q8N3X1, Q9P281, Q96ES7, Q03989, P51790, A4D1E9, Q6PHW0, Q9P2M1, Q96J77, Q562F6, Q14919, Q9UKZ4, Q8TBK6, Q14680, A0FGR9, Q9H159, Q92622, P41220, O14662, Q8N108, Q8IZF2, P02549, Q15149, P04275, Q14739, O14727, Q96BJ8, Q8N6M0, P11532 |

**Supplementary Table 15.** Analysis of physicochemical properties of binding interfaces of histone proteins and binding partners. Interfacial residues are categorized into five groups based on their physicochemical properties including polar-uncharged, nonpolar-aliphatic, nonpolar-aromatic, negatively charged, and positively charged.

| Type of binding interface | Type of interfacial residues | Residue name | Number of interfacial residues |
| --- | --- | --- | --- |
| Interfacial residues on histones | Polar-uncharged | S, T, C, P, N, Q | 1591 |
|  | Nonpolar-aliphatic | G, A, V, L, M, I | 2074 |
|  | Nonpolar-aromatic | F, Y, W | 296 |
|  | Negatively charged | D, E | 567 |
|  | Positively charged | K, R, H | 1777 |
| Interfacial residues on histone binding partners | Polar-uncharged | S, T, C, P, N, Q | 2865 |
|  | Nonpolar-aliphatic | G, A, V, L, M, I | 3419 |
|  | Nonpolar-aromatic | F, Y, W | 1854 |
|  | Negatively charged | D, E | 2250 |
|  | Positively charged | K, R, H | 1728 |

**Supplementary Table 16.** Number of histone-partner interactions associated with different types of histone post-translational modifications.

| Histone residue type | PTM type | Number of associated histone-partner interactions at residue level |
| --- | --- | --- |
| LYSINE | M3L(TRIMETHYL-LYSINE) | 1134 |
|  | ALY(ACETYL-LYSINE) | 882 |
|  | MLY(DIMETHYL-LYSINE) | 265 |
|  | MLZ(METHYL-LYSINE) | 182 |
|  | KCR(CROTONYL-LYSINE) | 167 |
| SERINE | SEP(PHOSPHOSERINE) | 92 |
| ARGININE | 2MR(DIMETHYL-ARGININE) | 66 |
|  | DA2(DIMETHYL-ARGININE) | 37 |
| THREONINE | TPO(PHOSPHOTHREONINE) | 61 |

**Supplementary Table 17.** Functional classification of histone/nucleosome binding proteins in human structural interactome using the PATHER^9^ protein class.

| PATHER protein class | Number of proteins |
| --- | --- |
| chromatin/chromatin-binding, or -regulatory protein (PC00077) | 52 |
| gene-specific transcriptional regulator (PC00264) | 17 |
| protein modifying enzyme (PC00260) | 14 |
| DNA metabolism protein (PC00009) | 12 |
| metabolite interconversion enzyme (PC00262) | 8 |
| defense/immunity protein (PC00090) | 6 |
| RNA metabolism protein (PC00031) | 6 |
| transporter (PC00227) | 3 |
| chaperone (PC00072) | 3 |
| scaffold/adaptor protein (PC00226) | 2 |

**Supplementary Table 18.**  Functional classification of histone/nucleosome binding proteins in human cross-linking interactome using the PATHER^9^ protein class.

| PATHER protein class | Number of proteins |
| --- | --- |
| RNA metabolism protein (PC00031) | 74 |
| cytoskeletal protein (PC00085) | 65 |
| translational protein (PC00263) | 62 |
| protein modifying enzyme (PC00260) | 59 |
| metabolite interconversion enzyme (PC00262) | 54 |
| gene-specific transcriptional regulator (PC00264) | 46 |
| scaffold/adaptor protein (PC00226) | 29 |
| DNA metabolism protein (PC00009) | 24 |
| chaperone (PC00072) | 23 |
| protein-binding activity modulator (PC00095) | 22 |
| chromatin/chromatin-binding, or -regulatory protein (PC00077) | 22 |
| transporter (PC00227) | 18 |
| membrane traffic protein (PC00150) | 16 |
| cell adhesion molecule (PC00069) | 10 |
| extracellular matrix protein (PC00102) | 6 |
| transmembrane signal receptor (PC00197) | 5 |
| defense/immunity protein (PC00090) | 4 |
| transfer/carrier protein (PC00219) | 4 |
| calcium-binding protein (PC00060) | 3 |
| structural protein (PC00211) | 3 |
| intercellular signal molecule (PC00207) | 2 |

**Supplementary Table 19.** Functional classification of histone/nucleosome binding proteins in human high-throughput interactome using the PATHER^9^ protein class.

| PATHER protein class | Number of proteins |
| --- | --- |
| protein modifying enzyme (PC00260) | 223 |
| gene-specific transcriptional regulator (PC00264) | 191 |
| RNA metabolism protein (PC00031) | 178 |
| metabolite interconversion enzyme (PC00262) | 132 |
| chromatin/chromatin-binding, or -regulatory protein (PC00077) | 119 |
| cytoskeletal protein (PC00085) | 86 |
| translational protein (PC00263) | 83 |
| protein-binding activity modulator (PC00095) | 78 |
| DNA metabolism protein (PC00009) | 76 |
| scaffold/adaptor protein (PC00226) | 69 |
| transporter (PC00227) | 53 |
| chaperone (PC00072) | 39 |
| transmembrane signal receptor (PC00197) | 32 |
| membrane traffic protein (PC00150) | 25 |
| defense/immunity protein (PC00090) | 24 |
| cell adhesion molecule (PC00069) | 16 |
| intercellular signal molecule (PC00207) | 14 |
| extracellular matrix protein (PC00102) | 11 |
| calcium-binding protein (PC00060) | 11 |
| structural protein (PC00211) | 10 |
| transfer/carrier protein (PC00219) | 6 |
| viral or transposable element protein (PC00237) | 5 |
| cell junction protein (PC00070) | 2 |

**Supplementary Table 20.** Identifications of hub nodes (MCC≥10) in human global histone structural interactome using MCC (Maximal Clique Centrality) scores.

| Protein Name | Uniprot ID | MCC | Degree |
| --- | --- | --- | --- |
| Polyubiquitin-B | P0CG47 | 187 | 118 |
| Maltose/maltodextrin-binding periplasmic protein | P0AEX9 | 112 | 111 |
| HLA class II histocompatibility antigen, DR alpha chain | P01903 | 92 | 57 |
| HLA class II histocompatibility antigen, DRB1 beta chain | P01911 | 74 | 39 |
| DNA-directed RNA polymerase subunit beta | C4QZQ7 | 68 | 25 |
| DNA-directed RNA polymerase subunit | C4R4Y0 | 65 | 22 |
| Transcription elongation factor SPT5 | C4R370 | 62 | 15 |
| Cellular tumor antigen p53 | P04637 | 50 | 44 |
| Ubiquitin-conjugating enzyme E2 D3 | P61077 | 44 | 12 |
| Ubiquitin-ribosomal protein eS31 fusion protein | P62979 | 30 | 27 |
| Bromodomain-containing protein 4 | O60885 | 29 | 28 |
| BRCA1-associated RING domain protein 1 | Q99728 | 28 | 7 |
| Envelope glycoprotein gp160 | Q2N0S6 | 26 | 26 |
| 14-3-3 protein zeta/delta | P63104 | 26 | 26 |
| Histone-lysine N-methyltransferase, H3 lysine-79 specific | Q8TEK3 | 24 | 13 |
| DNA replication licensing factor MCM2 | P49736 | 23 | 16 |
| E3 ubiquitin-protein ligase RING2 | Q99496 | 23 | 9 |
| DASH complex subunit DAD4 | G0S589 | 21 | 7 |
| WD repeat-containing protein 5 | P61964 | 21 | 17 |
| Vps72/YL1 C-terminal domain-containing protein | G0S590 | 20 | 6 |
| **RuvB-like helicase** | G0RYC2 | 19 | 6 |
| **TP53-binding protein 1** | Q12888 | 19 | 14 |
| **Breast cancer type 1 susceptibility protein** | P38398 | 18 | 10 |
| **Centromere protein C** | Q03188 | 18 | 13 |
| **Centromere protein N** | Q96H22 | 18 | 13 |
| **CREB-binding protein** | Q92793 | 17 | 15 |
| **Histone-lysine N-methyltransferase 2A** | Q03164 | 15 | 11 |
| **Transcription elongation factor Spt6** | C4R7H2 | 14 | 5 |
| **HLA class I histocompatibility antigen, C alpha chain** | P10321 | 13 | 11 |
| **Bromodomain-containing protein 3** | Q15059 | 13 | 12 |
| **Ubiquitin carboxyl-terminal hydrolase BAP1** | Q92560 | 12 | 7 |
| **FACT complex subunit** | C4QYQ8 | 12 | 8 |
| **Transcription initiation factor TFIID subunit 1** | P21675 | 12 | 12 |
| **Polycomb protein EED** | O75530 | 12 | 9 |
| **Histone chaperone ASF1A** | Q9Y294 | 12 | 7 |
| **Peptidyl-prolyl cis-trans isomerase E** | Q9UNP9 | 11 | 11 |
| **Protein that interacts with Spt6p and copurifies with Spt5p and RNA polymerase II** | C4R7L8 | 11 | 6 |
| **Transportin-1** | Q92973 | 10 | 10 |
| **Histone-lysine N-methyltransferase EZH2** | Q15910 | 10 | 7 |
| **Speckle-type POZ protein** | O43791 | 10 | 10 |
| **RuvB-like helicase** | G0RYC2 | 19 | 6 |

**Supplementary Table 21.** Identifications of hub nodes (MCC≥40) in human global histone cross-linking interactome using MCC (Maximal Clique Centrality) scores.

| Protein Name | Uniprot ID | MCC | Degree |
| --- | --- | --- | --- |
| **Heat shock 70 kDa protein 1A** | P0DMV8 | 247 | 149 |
| **Heat shock protein HSP 90-beta** | P08238 | 215 | 92 |
| **Ubiquitin-ribosomal protein eS31 fusion protein** | P62979 | 1133 | 43 |
| **Alpha-enolase** | P06733 | 247 | 109 |
| **Heterogeneous nuclear ribonucleoproteins A2/B1** | P22626 | 425 | 54 |
| **Ubiquitin-ribosomal protein eL40 fusion protein** | P62987 | 1146 | 39 |
| **Titin** | Q8WZ42 | 517 | 53 |
| **Non-histone chromosomal protein HMG-17** | P05204 | 144 | 50 |
| **Glyceraldehyde-3-phosphate dehydrogenase** | P04406 | 112 | 70 |
| **Large ribosomal subunit protein eL8** | P62424 | 89 | 36 |
| **Trinucleotide repeat-containing gene 18 protein** | O15417 | 327 | 26 |
| **Histone-lysine N-methyltransferase 2A** | Q03164 | 416 | 33 |
| **Large ribosomal subunit protein eL14** | P50914 | 71 | 33 |
| **Putative elongation factor 1-alpha-like 3** | Q5VTE0 | 111 | 57 |
| **Endoplasmin** | P14625 | 65 | 30 |
| **Large ribosomal subunit protein uL23** | P62750 | 69 | 27 |
| **High mobility group protein HMG-I/HMG-Y** | P17096 | 295 | 29 |
| **Nebulin** | P20929 | 76 | 24 |
| **Endoplasmic reticulum chaperone BiP** | P11021 | 74 | 41 |
| **Nucleolin** | P19338 | 46 | 29 |
| **Large ribosomal subunit protein eL36** | Q9Y3U8 | 56 | 21 |
| **Heterogeneous nuclear ribonucleoprotein U** | Q00839 | 71 | 32 |
| **Small ribosomal subunit protein uS10** | P60866 | 48 | 24 |
| **Neurofilament medium polypeptide** | P07197 | 60 | 36 |
| **ADP/ATP translocase 3** | P12236 | 120 | 16 |
| **Large ribosomal subunit protein eL19** | P84098 | 47 | 24 |
| **Heterogeneous nuclear ribonucleoprotein A1** | P09651 | 57 | 20 |
| **Small glutamine-rich tetratricopeptide repeat-containing protein alpha** | O43765 | 179 | 16 |
| **Large ribosomal subunit protein eL24** | P83731 | 44 | 20 |
| **Bifunctional glutamate/proline--tRNA ligase** | P07814 | 75 | 19 |
| **Large ribosomal subunit protein uL4** | P36578 | 43 | 32 |
| **Non-histone chromosomal protein HMG-14** | P05114 | 57 | 18 |
| **Structural maintenance of chromosomes protein 1A** | Q14683 | 48 | 14 |
| **Small nuclear ribonucleoprotein Sm D2** | P62316 | 107 | 15 |
| **Small ribosomal subunit protein eS25** | P62851 | 42 | 20 |
| **Small ribosomal subunit protein eS7** | P62081 | 46 | 32 |
| **Threonine--tRNA ligase 1, cytoplasmic** | P26639 | 45 | 28 |
| **GTP-binding nuclear protein Ran** | P62826 | 45 | 22 |
| **Dynein axonemal heavy chain 6** | Q9C0G6 | 43 | 14 |
| **High mobility group protein B1** | P09429 | 45 | 23 |
| **Protein disulfide-isomerase A3** | P30101 | 56 | 26 |
| **Neuroblast differentiation-associated protein AHNAK** | Q09666 | 43 | 14 |
| **Elongation factor 1-alpha 1** | P68104 | 43 | 18 |
| **ATP-binding cassette sub-family F member 2** | Q9UG63 | 45 | 12 |
| **Nuclear mitotic apparatus protein 1** | Q14980 | 49 | 12 |
| **Pleckstrin homology-like domain family A member 2** | Q53GA4 | 100 | 8 |
| **Small ribosomal subunit protein uS19** | P62841 | 42 | 37 |

**Supplementary Table 22.** Functional classification of hub nodes in human global histone structural interactome using the PATHER protein class^9^.

| PATHER protein class | Number of proteins |
| --- | --- |
| chromatin/chromatin-binding, or -regulatory protein (PC00077) | 7 |
| protein modifying enzyme (PC00260) | 5 |
| defense/immunity protein (PC00090) | 3 |
| transporter (PC00227) | 2 |
| DNA metabolism protein (PC00009) | 2 |
| scaffold/adaptor protein (PC00226) | 1 |
| RNA metabolism protein (PC00031) | 1 |
| gene-specific transcriptional regulator (PC00264) | 1 |

**Supplementary Table 23.** Functional classification of hub nodes in human global histone cross-linking interactome using the PATHER protein class^9^.

| PATHER protein class | Number of proteins |
| --- | --- |
| Translational protein (PC00263) | 16 |
| Chaperone (PC00072) | 5 |
| RNA metabolism protein (PC00031) | 4 |
| cytoskeletal protein (PC00085) | 3 |
| gene-specific transcriptional regulator (PC00264) | 3 |
| chromatin/chromatin-binding, or -regulatory protein (PC00077) | 3 |
| metabolite interconversion enzyme (PC00262) | 2 |
| protein-binding activity modulator (PC00095) | 1 |
| transfer/carrier protein (PC00219) | 1 |
| structural protein (PC00211) | 1 |

**Supplementary Table 24**. Classifications of 83 representative nucleosome complex structures by their binding modes.

| Binding Mode Types | PDB IDs |
| --- | --- |
| Partners only bind to DNA | \| 7U50, 7XZZ, 8ATF, 7ZS9, 8G8G, 6T7C, 7SCZ, 8H0V, 6R25, 5X0X, 6YOV, 6T7B, 6T90, 6R91, 6X0N, 6USJ, 6R90 \| \| --- \| |
| Partners only bind to histone proteins | \| 5KGF, 5MLU, 5E5A, 6E0P, 5GTC, 7EG6, 7LYB, 6T9L, 7Y7I, 7D1Z, 7E9F, 7TAN \| \| --- \| |
| Partners bind both DNA and histone proteins | \| 3TU4, 7CCQ, 7W9V, 8GPN, 6WKR, 8F86, 7ENN, 6FTX, 8AV6, 6KIU, 6PWX, 5X0Y, 6LTJ, 6G0L, 6PWF, 6PWV, 8DU4, 6NE3, 8H1T, 6RYU, 6JYL, 6KIW, 3MVD, 7EGP, 7LYC, 6VEN, 7UV9, 7E8D, 7ZSA, 6GEJ, 7SSA, 6PA7, 6S01, 7CRQ, 6TDA, 7OHA, 6QLD, 7XCT, 7PH6, 7VVU, 6KW3, 7OH9, 7YWX, 7BWD, 6VYP, 7Y8R, 6NZO, 7XD0, 6MUP  6Z6P, 6R1U, 8GRM, 7EA8, 6R8Z \| \| --- \| |

**Supplementary Table 25.**  Mapping of partner binding hotspots and cancer mutation hotspots onto histone consensus sequences. The consensus sequences were taken from the alignment of histone sequences for each histone type (Supplementary Figure 2-6). The partner binding hotspots are defined as the histone residues in consensus sequences which interact with at least three different binding partners. The cancer mutation hotspots are defined as the histone residues in consensus sequences that carry at least five different cancer mutations in *combined histone cancer mutation set*. Then, overlapping ratios are calculated as the number of overlapped partner hotspots (overlapped with any mutation hotspots) divided by the total number of partner hotspots.

| Histone type | Number of partner binding hotspots | Number of cancer mutation hotspots | Overlapping ratio |
| --- | --- | --- | --- |
| H2A | 31 | 106 | 0.81 |
| H2B | 32 | 106 | 0.81 |
| H3 | 21 | 102 | 0.81 |
| H4 | 20 | 40 | 0.50 |

**Supplementary Table 26.**  Mapping of post-translational modification (PTM) sites and histone cancer mutation hotspots onto histone consensus sequences. The consensus sequences were taken from the alignment of histone sequences for each histone type (Supplementary Figure 2-6). The cancer mutation hotspots are defined as the histone residues in consensus sequences which carry at least five different cancer mutations in *combined histone cancer mutation set*. Then, overlapping ratios are calculated as the number of overlapped PTM sites (overlapped with any mutation hotspots) divided by the total number of PTM sites.

| Histone type | Number of PTM sites | Number of cancer mutation hotspots | Overlapping ratio |
| --- | --- | --- | --- |
| H1 | 1 | 97 | 0 |
| H2A | 9 | 106 | 0.56 |
| H2B | 0 | 106 | 0 |
| H3 | 29 | 102 | 0.69 |
| H4 | 14 | 40 | 0.57 |

**Supplementary Table 27.** Comparison of binding free energy changes (∆∆Gs) caused by histone cancer mutations among different histone types and interaction types. Tukey HSD tests were performed to compare the differences of ∆∆G value distributions and the null hypothesis is that the mean values of ∆∆Gs in two groups are equal. The *combined histone cancer mutation set* were mapped onto the binding interfaces of each histone interaction type. The ∆∆Gs were calculated for each mutation and used for the comparison between different groups.

| Type of interaction | group1 | group2 | Mean diff | p-adj | lower | upper | reject |
| --- | --- | --- | --- | --- | --- | --- | --- |
| Histone-DNA | H1 | H2A | 0.41 | <1.0E-10 | 0.33 | 0.48 | TRUE |
|  | H1 | H2B | 0.09 | 8.90E-03 | 0.02 | 0.17 | TRUE |
|  | H1 | H3 | 0.35 | <1.0E-10 | 0.28 | 0.43 | TRUE |
|  | H1 | H4 | 0.48 | <1.0E-10 | 0.41 | 0.56 | TRUE |
|  | H2A | H2B | -0.31 | <1.0E-10 | -0.34 | -0.29 | TRUE |
|  | H2A | H3 | -0.05 | <1.0E-10 | -0.07 | -0.03 | TRUE |
|  | H2A | H4 | 0.08 | <1.0E-10 | 0.06 | 0.10 | TRUE |
|  | H2B | H3 | 0.26 | <1.0E-10 | 0.24 | 0.28 | TRUE |
|  | H2B | H4 | 0.39 | <1.0E-10 | 0.37 | 0.41 | TRUE |
|  | H3 | H4 | 0.13 | <1.0E-10 | 0.12 | 0.14 | TRUE |
| Histone-histone | H2A | H2B | -0.03 | <1.0E-10 | -0.04 | -0.02 | TRUE |
|  | H2A | H3 | -0.01 | 1.11E-01 | -0.02 | 0.00 | FALSE |
|  | H2A | H4 | 0.02 | <1.0E-10 | 0.02 | 0.03 | TRUE |
|  | H2B | H3 | 0.02 | <1.0E-10 | 0.01 | 0.03 | TRUE |
|  | H2B | H4 | 0.05 | <1.0E-10 | 0.05 | 0.06 | TRUE |
|  | H3 | H4 | 0.03 | <1.0E-10 | 0.03 | 0.04 | TRUE |
| Histone-partner | H1 | H2A | 0.48 | 4.90E-03 | 0.10 | 0.85 | TRUE |
|  | H1 | H2B | 0.42 | 2.16E-02 | 0.04 | 0.80 | TRUE |
|  | H1 | H3 | 0.21 | 5.61E-01 | -0.17 | 0.58 | FALSE |
|  | H1 | H4 | 0.31 | 1.50E-01 | -0.06 | 0.69 | FALSE |
|  | H2A | H2B | -0.06 | 2.40E-01 | -0.14 | 0.02 | FALSE |
|  | H2A | H3 | -0.27 | <1.0E-10 | -0.32 | -0.22 | TRUE |
|  | H2A | H4 | -0.16 | <1.0E-10 | -0.22 | -0.11 | TRUE |
|  | H2B | H3 | -0.21 | <1.0E-10 | -0.28 | -0.15 | TRUE |
|  | H2B | H4 | -0.11 | 1.00E-04 | -0.17 | -0.04 | TRUE |
|  | H3 | H4 | 0.11 | <1.0E-10 | 0.08 | 0.14 | TRUE |

**Supplementary Table 28.** Comparison of binding free energy changes (∆∆Gs) caused by histone cancer mutations among different histone types and interaction types. Tukey HSD tests were performed to compare the differences of ∆∆G value distributions and the null hypothesis is that the mean values of ∆∆Gs in two groups are equal. The *refined histone cancer mutation set* were mapped onto the binding interfaces of each histone interaction type. The ∆∆Gs were calculated for each mutation and used for the comparison between different groups.

| Type of interaction | group1 | group2 | Mean diff | p-adj | lower | upper | reject |
| --- | --- | --- | --- | --- | --- | --- | --- |
| Histone-DNA | H1 | H2A | 0.85 | <1.0E-10 | 0.56 | 1.13 | TRUE |
|  | H1 | H2B | -0.14 | 5.19E-01 | -0.38 | 0.10 | FALSE |
|  | H1 | H3 | 0.50 | <1.0E-10 | 0.26 | 0.73 | TRUE |
|  | H1 | H4 | 0.73 | <1.0E-10 | 0.49 | 0.96 | TRUE |
|  | H2A | H2B | -0.99 | <1.0E-10 | -1.17 | -0.80 | TRUE |
|  | H2A | H3 | -0.35 | <1.0E-10 | -0.52 | -0.18 | TRUE |
|  | H2A | H4 | -0.12 | 3.07E-01 | -0.29 | 0.05 | FALSE |
|  | H2B | H3 | 0.64 | <1.0E-10 | 0.56 | 0.71 | TRUE |
|  | H2B | H4 | 0.86 | <1.0E-10 | 0.79 | 0.94 | TRUE |
|  | H3 | H4 | 0.23 | <1.0E-10 | 0.19 | 0.27 | TRUE |
| Histone-histone | H2A | H2B | -0.27 | <1.0E-10 | -0.37 | -0.16 | TRUE |
|  | H2A | H3 | 0.00 | 1.00E+00 | -0.10 | 0.10 | FALSE |
|  | H2A | H4 | -0.25 | <1.0E-10 | -0.35 | -0.15 | TRUE |
|  | H2B | H3 | 0.26 | <1.0E-10 | 0.23 | 0.30 | TRUE |
|  | H2B | H4 | 0.02 | 6.08E-01 | -0.02 | 0.06 | FALSE |
|  | H3 | H4 | -0.25 | <1.0E-10 | -0.26 | -0.23 | TRUE |
| Histone-partner | H2A | H2B | 0.44 | 3.11E-02 | 0.03 | 0.85 | TRUE |
|  | H2A | H3 | -0.24 | 3.41E-01 | -0.60 | 0.13 | FALSE |
|  | H2A | H4 | -0.22 | 4.25E-01 | -0.59 | 0.15 | FALSE |
|  | H2B | H3 | -0.68 | <1.0E-10 | -0.88 | -0.48 | TRUE |
|  | H2B | H4 | -0.66 | <1.0E-10 | -0.86 | -0.45 | TRUE |
|  | H3 | H4 | 0.02 | 9.04E-01 | -0.05 | 0.09 | FALSE |

**Supplementary Table 29.** Recurrent histone cancer mutations with predicted strong disruptive effects on histone-DNA interactions. The frequency of mutations represents the number of times observed in patients in the *refined histone cancer mutation set.* Mean ΔΔG was calculated with top three nucleosome structures with largest binding free energy changes.

| Cancer mutation | Frequency | Uniprot ID | Histone Gene | Mean ΔΔG (kcal/mol) |
| --- | --- | --- | --- | --- |
| H4 K31N | 3 | P62805 | H4C8, H4C11 | 2.60 |
| H2A R29P | 3 | P0C0S8 | H2AC15, H2AC16, H2AC13 | 2.35 |
| H4 K79N | 4 | P62805 | H4C16, H4C5, H4C2, H4C12 | 2.42 |
| H2A R17P | 4 | P0C0S8 | H2AC16, H2AC17 | 2.20 |
| H3 R72P | 3 | P68431 | H3C2 | 2.20 |
| H3 R116P | 4 | P68431 | H3C4, H3C7, H3C8, H3C2 | 2.02 |
| H3 R83C | 5 | P68431 | H3C10, H3C12, H3C8, H3C2 | 1.99 |
| H3 R40C | 3 | P68431 | H3C8, H3C3, H3C12 | 1.99 |
| H4 R19C | 3 | P62805 | H4C13, H4C5 | 1.87 |
| H3 K36M | 23 | P84243, P68431 | H3C6, H3C3, H3C8, H3C4, H3C11, H3-3A, H3C10, H3-3B | 1.81 |
| H3 R53H | 4 | P68431 | H3C3, H3C8, H3C2 | 1.75 |
| H3 F67L | 4 | P68431 | H3C4, H3C3, H3C12 | 1.69 |
| H3 R63H | 3 | P68431 | H3C7, H3C12 | 1.62 |
| H4 R45Q | 3 | P62805 | H4C2 | 1.61 |
| H3 R116Q | 3 | P68431 | H3C2 | 1.48 |
| H3 E50D | 4 | P68431 | H3C2 | 1.47 |
| H3 K79N | 4 | P68431 | H3C4, H3C11, H3C6 | 1.47 |
| H3 R52C | 3 | Q16695 | H3-4 | 1.35 |
| H1 K74N | 3 | P10412 | H1-4 | 1.31 |
| H2A R31L | 4 | Q71UI9 | H2AZ2 | 1.26 |
| H3 Y41C | 5 | P84243 | H3-3B | 1.17 |
| H3 M120I | 3 | P68431 | H3C3, H3C7, H3C2 | 1.08 |
| H4 A33T | 4 | P62805 | H4C3, H4C8 | 1.02 |

**Supplementary Table 30.** Recurrent histone cancer mutations with predicted strong disruptive effects on histone-histone interactions. The frequency of mutations represents the number of times observed in patients in the *refined histone cancer mutation set.* Mean ΔΔG was calculated with top three nucleosome structures with largest binding free energy changes.

| Cancer mutation | Frequency | Uniprot ID | Histone Gene | Mean ΔΔG (kcal/mol) |
| --- | --- | --- | --- | --- |
| H3 R72P | 3 | P68431 | H3C2 | 3.64 |
| H3 R131P | 4 | P68431 | H3C11, H3C12 | 3.45 |
| H3 R116P | 4 | P68431 | H3C4, H3C7, H3C8, H3C2 | 3.16 |
| H2A R29P | 3 | P0C0S8 | H2AC15, H2AC16, H2AC13 | 2.83 |
| H2A R17P | 4 | P0C0S8 | H2AC16, H2AC17 | 2.68 |
| H4 R40C | 3 | P62805 | H4C9, H4C13, H4C14 | 2.17 |
| H4 K31N | 3 | P62805 | H4C8, H4C11 | 1.89 |
| H3 R63H | 3 | P68431 | H3C7, H3C12 | 1.99 |
| H4 D85N | 3 | P62805 | H4C2, H4C13, H4C5 | 1.90 |
| H4 K91N | 4 | P62805 | H4C5 | 1.73 |
| H3 F67L | 4 | P68431 | H3C4, H3C3, H3C12 | 1.85 |
| H3 E50Q | 4 | P68431 | H3C2 | 1.84 |
| H4 R45Q | 3 | P62805 | H4C2 | 1.81 |
| H3 Q55E | 4 | P68431 | H3C11, H3C8 | 1.79 |
| H4 E63Q | 3 | P62805 | H4C16, H4C12 | 1.79 |
| H4 K79N | 4 | P62805 | H4C16, H4C5, H4C2, H4C12 | 1.65 |
| H3 E94K | 6 | P68431 | H3C4, H3C6, H3C7, H3C2 | 1.77 |
| H3 D106N | 3 | P68431 | H3C3, H3C2 | 1.55 |
| H4 R19C | 3 | P62805 | H4C13, H4C5 | 1.72 |
| H3 E73K | 10 | P68431 | H3C10, H3C3, H3C8, H3C2 | 1.71 |
| H2B F70L | 11 | Q5QNW6, Q16778 | H2BC21, H2BC18 | 1.71 |
| H4 G42R | 6 | P62805 | H4C8 | 1.71 |
| H3 R83C | 5 | P68431 | H3C10, H3C12, H3C8, H3C2 | 1.71 |
| H3 E105Q | 13 | P68431 | H3C11, H3C6, H3C2 | 1.68 |
| H3 E97K | 16 | P68431 | H3C6, H3C3, H3C12, H3C4, H3C11, H3C10, H3C2 | 1.64 |
| H2B H49L | 3 | P62807 | H2BC6 | 1.66 |
| H3 Q55H | 6 | P68431 | H3C6, H3C7, H3C12, H3C11, H3C1 | 1.62 |
| H3 E73Q | 5 | P68431 | H3C11, H3C6, H3C3, H3C2 | 1.58 |
| H3 S87L | 3 | P68431 | H3C7, H3C12 | 1.51 |
| H3 E105K | 17 | P68431, P84243, Q71DI3 | H3C6, H3C7, H3C8, H3C4, H3C11, H3C13, H3-3B, H3C2 | 1.56 |
| H2B G60D | 3 | P62807 | H2BC4 | 1.56 |
| H3 E50D | 4 | P68431 | H3C2 | 1.54 |
| H3 R116Q | 3 | P68431 | H3C2 | 1.53 |
| H4 D68N | 4 | P62805 | H4C5, H4C12, H4C4 | 1.52 |

**Supplementary Table 31.** Recurrent histone cancer mutations with predicted strong disruptive effects on histone-partner interactions. The frequency of mutations represents the number of times observed in patients in the *refined histone cancer mutation set.* Mean ΔΔG was calculated with top three histone nucleosome complex structures with largest binding free energy changes.

| Cancer mutation | Frequency | Uniprot ID | Histone Gene | Mean ΔΔG (kcal/mol) |
| --- | --- | --- | --- | --- |
| H3 R26P | 5 | P68431 | H3C4, H3C2 | 3.08 |
| H2B H49L | 3 | P62807 | H2BC6 | 3.06 |
| H3 R131P | 4 | P68431 | H3C11, H3C12 | 2.65 |
| H3 R2C | 3 | P68431 | H3C4, H3C7, H3C1 | 2.44 |
| H3 Y41C | 5 | P84243 | H3-3B | 2.43 |
| H3 R116P | 4 | P68431 | H3C4, H3C7, H3C8, H3C2 | 2.36 |
| H4 R3G | 3 | P62805 | H4C2, H4C3, H4C11 | 2.16 |
| H3 R72P | 3 | P68431 | H3C2 | 2.13 |
| H3 R2H | 3 | P68431 | H3C10, H3C8 | 2.04 |
| H3 R2G | 3 | P84243 | H3-3B, H3-3A | 2.03 |
| H4 R19C | 3 | P62805 | H4C13, H4C5 | 2.00 |
| H4 E53D | 3 | P62805 | H4C2, H4C13, H4C5 | 2.00 |
| H3 R40C | 3 | P68431 | H3C8, H3C3, H3C12 | 1.95 |
| H4 E63Q | 3 | P62805 | H4C16, H4C12 | 1.95 |
| H3 K27M | 28 | P84243, P68431 | H3C7, H3C3, H3C4, H3-3A, H3C2 | 1.64 |
| H4 R3C | 9 | P62805 | H4C4, H4C11, H4C13, H4C2, H4C12 | 1.86 |
| H4 R40C | 3 | P62805 | H4C9, H4C13, H4C14 | 1.78 |
| H4 D85N | 3 | P62805 | H4C2, H4C13, H4C5 | 1.72 |
| H3 R26Q | 3 | P68431 | H3C10, H3C4 | 1.67 |
| H3 R26H | 3 | P68431 | H3C11, H3C6, H3C2 | 1.62 |
| H3 K4M | 8 | P84243, P68431 | H3C11, H3-3A, H3C1, H3C10, H3-3B | 1.54 |
| H2B E113K | 6 | P06899, P62807 | H2BC11, H2BC8, H2BC4 | 1.53 |
| H2A E121Q | 4 | P20671 | H2AC7 | 1.50 |

**References**:

1 research, U. C. J. N. a. UniProt: a worldwide hub of protein knowledge. **47**, D506-D515 (2019).

2 Draizen, E. J. *et al.* HistoneDB 2.0: a histone database with variants—an integrated resource to explore histones and their variants. **2016**, baw014 (2016).

3 Madeira, F. *et al.* Search and sequence analysis tools services from EMBL-EBI in 2022. *Nucleic Acids Res* **50**, W276-W279, doi:10.1093/nar/gkac240 (2022).

4 Gao, J. *et al.* Integrative analysis of complex cancer genomics and clinical profiles using the cBioPortal. **6**, pl1-pl1 (2013).

5 Nacev, B. A. *et al.* The expanding landscape of ‘oncohistone’mutations in human cancers. **567**, 473-478 (2019).

6 Burley, S. K. *et al.* Protein Data Bank (PDB): the single global macromolecular structure archive. 627-641 (2017).

7 Armeev, G. A., Gribkova, A. K. & Shaytan, A. K. J. b. NucleosomeDB-a database of 3D nucleosome structures and their complexes with comparative analysis toolkit. 2023.2004. 2017.537230 (2023).

8 Gao, J. *et al.* Integrative analysis of complex cancer genomics and clinical profiles using the cBioPortal. *Science signaling* **6**, pl1-pl1 (2013).

9 Thomas, P. D. *et al.* PANTHER: a library of protein families and subfamilies indexed by function. **13**, 2129-2141 (2003).
